# Cardiac and Respiratory Patterns Synchronize between Persons during Choir Singing

**DOI:** 10.1371/journal.pone.0024893

**Published:** 2011-09-21

**Authors:** Viktor Müller, Ulman Lindenberger

**Affiliations:** Center for Lifespan Psychology, Max Planck Institute for Human Development, Berlin, Germany; Humboldt University, Germany

## Abstract

Dyadic and collective activities requiring temporally coordinated action are likely to be associated with cardiac and respiratory patterns that synchronize within and between people. However, the extent and functional significance of cardiac and respiratory between-person couplings have not been investigated thus far. Here, we report interpersonal oscillatory couplings among eleven singers and one conductor engaged in choir singing. We find that: (a) phase synchronization both in respiration and heart rate variability increase significantly during singing relative to a rest condition; (b) phase synchronization is higher when singing in unison than when singing pieces with multiple voice parts; (c) directed coupling measures are consistent with the presence of causal effects of the conductor on the singers at high modulation frequencies; (d) the different voices of the choir are reflected in network analyses of cardiac and respiratory activity based on graph theory. Our results suggest that oscillatory coupling of cardiac and respiratory patterns provide a physiological basis for interpersonal action coordination.

## Introduction

As noted by Glass ([1], p. 277), “complex bodily rhythms are ubiquitous in living organisms”. This leads to the question how these rhythms interact with each other and the external environment [1], especially if this environment is extended to interacting people [2], [3]. Singing in a choir is a highly synchronized form of social interaction that presumably mandates synchronization of bodily activity. In particular, synchronized breathing may have a coordinating function in choir performance. The organizing role of collectively synchronized cardiac and respiratory activity during choir performance has not yet been investigated quantitatively, though appropriate quantitative measures for assessing such synchronization phenomena are available. Changes in heart rate variability (HRV) induced by respiration or breathing [4], [5], [6] are known as respiratory sinus arrhythmia (RSA). As reported by Bonsignore et al. [7] (see also [8]), heart rate (HR) is known to increase at inspiration and decrease at expiration. Schäfer et al. [9] found that respiration and cardiac rhythms are weakly coupled within persons, with a driving effect of respiration on HRV. In a study using a paced breathing protocol, Censi et al. [10] found that synchronization patterns between respiration and cardiovascular variability differ at different breathing rhythms and are transient rather than permanent phenomena. In a study with resting human subjects, Tzeng et al. [11] found cardioventilatory coupling at both low (0.04–0.15 Hz) and high (0.15–0.40 Hz) frequency ranges. Recently, Van Leeuwen et al. [12] found that synchronization between fetal and maternal cardiac activity increased at higher paced breathing rates and occurred at specific time points of the maternal breathing cycle.

RSA, or the synchronization between respiration and HRV, has mostly been investigated at rest in a supine position [11], [13] or under steady-state conditions, when metabolic activity and autonomic tone are largely constant [4]. Pomeranz et al. [14] reported fewer high-frequency fluctuations of the HRV when standing relative to a supine position, which is consistent with the claim that such fluctuations are mediated solely by the parasympathetic nervous system. Data on RSA during singing or similar performance conditions are scarce or lacking [15], [16], [17], [18]. In the study reported by Grape et al. [17], professional singers showed an increase in the low- and the high-frequency spectral power of HRV during singing, whereas amateur singers did not show such changes. Except for the study on maternal and fetal cardiac activity by Van Leeuwen et al. [12], synchronization patterns of respiration or HRV between individuals have not yet been investigated.

In the current study, we obtained simultaneous ECG and respiration measures from a conductor and eleven singers comprising a choir. We computed indicators of phase synchronization and directional coupling to investigate synchronization mechanisms in individuals involved in singing a four-part song and a three-part canon, each sung either in multiple voice parts or in unison (using one entry of the canon, or the soprano part of the song, which contained the song's melody). In addition to being sung with eyes open, the canon was also sung with eyes closed to compare normal singing to singing without the visual cues provided by the conductor. In this condition, the conductor sang along with the third entry of the canon. To obtain baseline measures, the members of the choir were also assessed in rest conditions before and after singing. In addition to synchronization or coupling measures, we used graph-theoretical tools to describe quantitatively the network structure and network dynamics of the choir under different singing conditions.

Our goal in this present paper is to explore the degree of between-person synchronicity at the autonomic nervous-system level during coordinated behaviour and to unveil the specific group dynamics underlying such complex interaction as choral singing. Our expectations were that synchronization between choir members for both respiration and HRV would be higher during singing than during rest and higher during singing in unison than during singing a part song or canon. We also expected that it would be possible to divide the choir members into groups or modules depending on the part they sang in the choir, using modularity measures derived from a graph-theoretic approach [19], [20], [21].

## Results

### Power Spectral Density (PSD)

**Figure 1:**
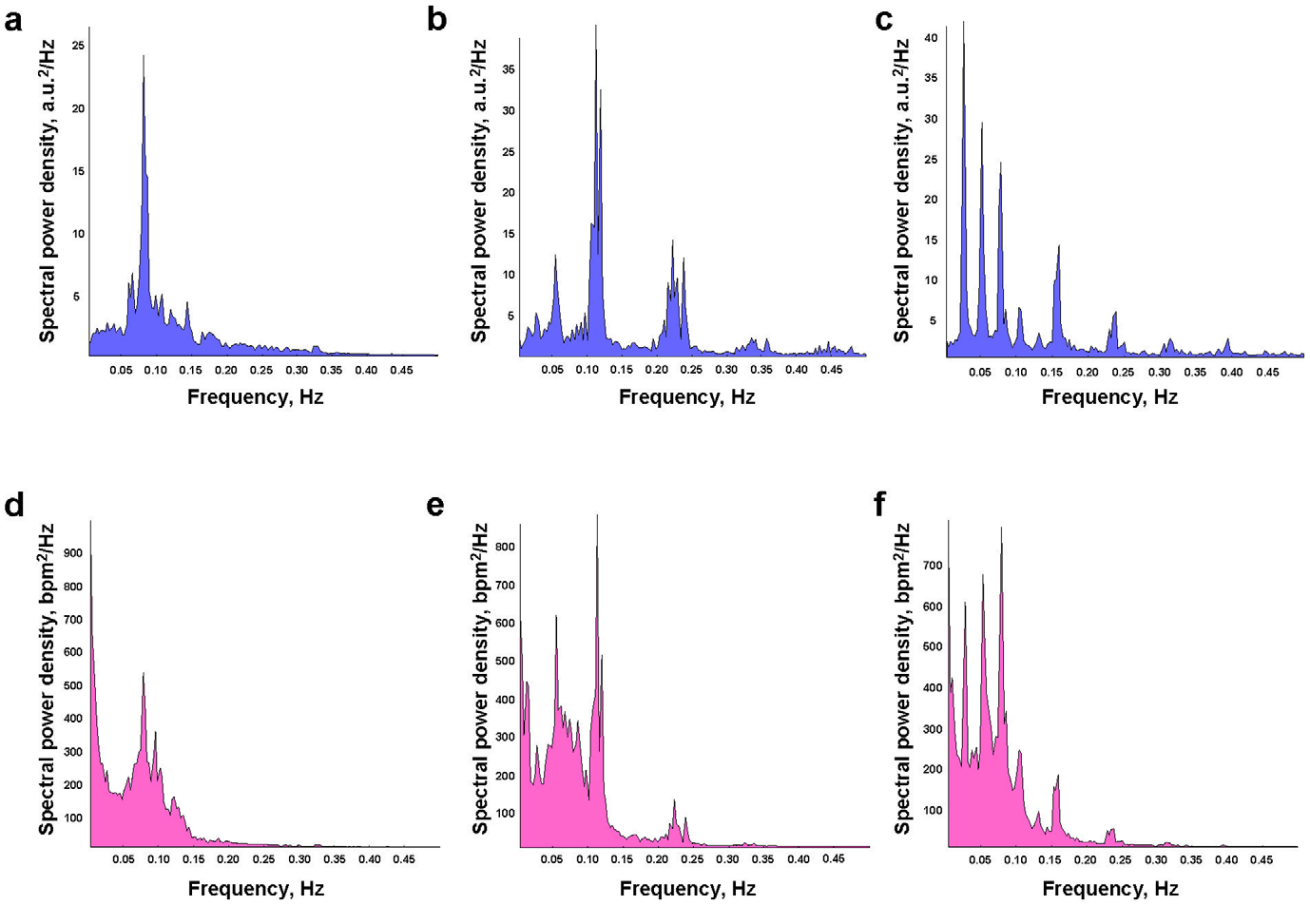
Spectral power density (SPD) for respiration and HRV time series during rest and singing conditions. **a-c**, SPD for respiration during rest (**a**), song singing (**b**) and canon singing (**c**); **d-f**, SPD for HRV during rest (**d**), song singing (**e**), and canon singing (**f**). Note that SPD was averaged across subjects and all respective conditions.

After preprocessing the data and calculating the heart rate (HR) from the ECG (see Methods), we determined, as a first step, the PSD (Power Spectral Density) in the corresponding time series for each participant under the different conditions. Below, grand averages of the PSD data for respiration and HRV are shown separately for rest and singing (both song and canon) conditions (see Figure 1). During rest, the PSD peaks were in a lower frequency range (between 0.06 and 0.15 Hz) than commonly reported in the literature [4], [6], [22]. Singing conditions showed at least three prominent peaks while singing the song (0.05, 0.11, and 0.24 Hz), and six for singing the canon (0.03, 0.05, 0.08, 0.11, 0.16, and 0.24 Hz). These frequencies represent harmonics with a basic frequency of 0.027 Hz. All of the peaks found in respiration could also be seen in the HRV PSD. In sum, these initial analyses revealed that the RSA frequency range of interest needed to be extended to lower frequency ranges down to 0.03 Hz, at least for singing conditions.

### Synchronization patterns and synchronization indices

**Figure 2:**
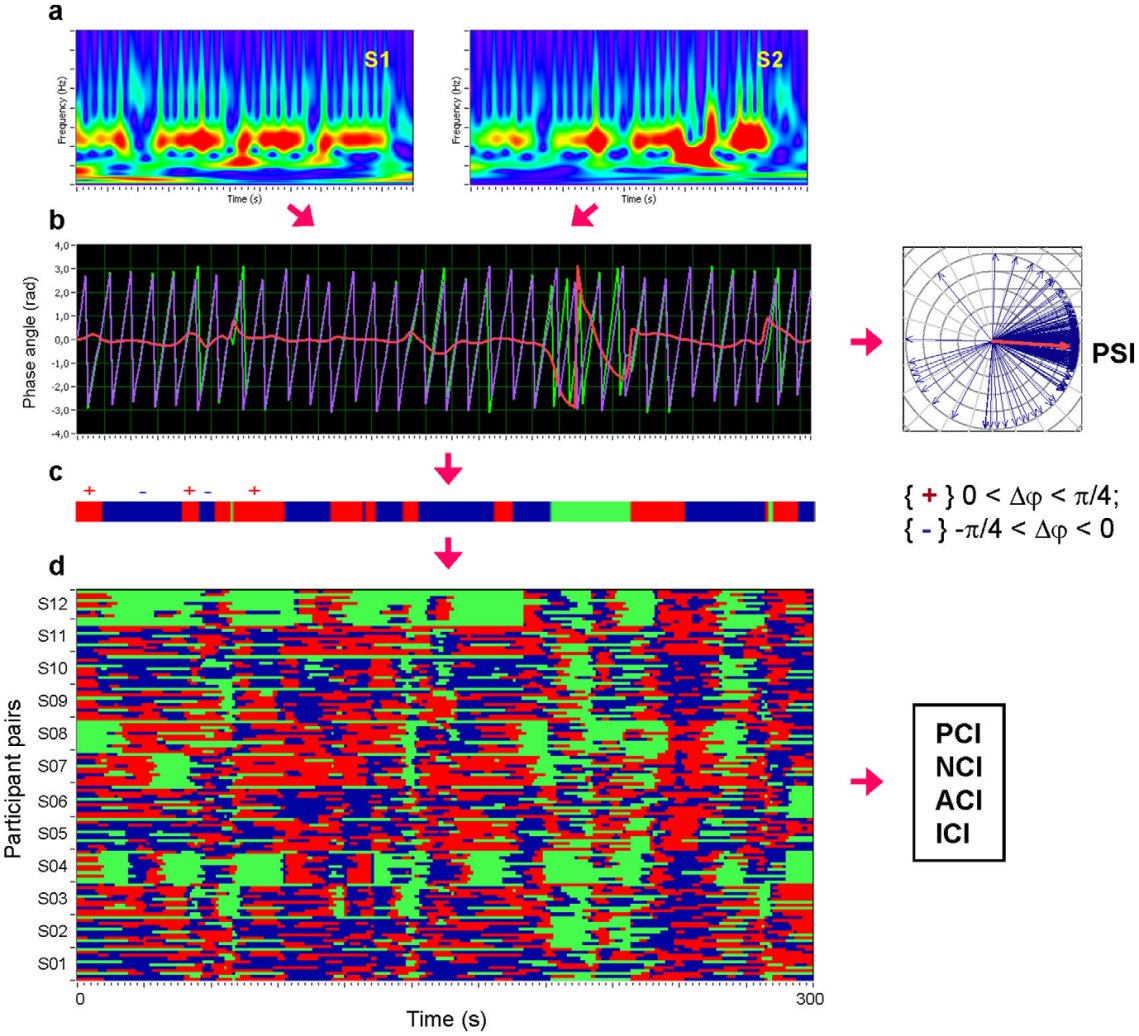
Schematic presentation of phase synchronization analyses. **a**, Complex Morlet wavelet transformation of signals from two subjects (S1 and S2) in the time-frequency domain. **b**, Time course of instantaneous phases from two subjects and their phase difference (S1  =  violet curve; S2  =  green curve; S1-S2  =  red curve); at the right, the phase difference is depicted in form of the vectors in complex space, where the blue arrows reflect single phase angles and the red arrow represents the mean vector of the angular dispersions; its length displays the *PSI* measure. **c**, Coding of the phase difference (-π/4<S1-S2<0: blue stripes; 0<S1-S2<+π/4: red stripes; S1-S2<-π/4 or S1-S2 > +π/4: green stripes  =  non-synchronization). **d**, Pair-wise synchronization pattern of the whole choir (132 lines). Each line represents one pair of subjects. For each subject, 11 lines represent the coded phase difference between this subject and other choir participants. Note that these lines or phase differences coded with +1 (red), 0 (green) or -1 (blue) at each time point are used for calculation of the four synchronization measures (i.e., *PCI*, *NCI*, *ACI*, and *ICI*) as described in the Methods section.

Next, we used complex wavelet transforms to determine the instantaneous phases of the respiration and HRV measures and the instantaneous phase differences between all possible pairs of participants. To determine for how long phase differences remained constant, we calculated the Phase Synchronization Index (*PSI*), defined as the mean vector length of the angular dispersions of the phase difference in a complex space. Afterwards, we ascertained how long phase differences remained stable within pre-defined phase angle boundaries. We looked for in-phase synchronizations with phase angles spread between -π/4 and + π/4 (cf. [23]). We also distinguished between positive and negative deviations from phase zero. As depicted in Figure 2, we marked negative deviations in the range between - π/4 and 0 in blue (coded with “-1”) and the positive deviations in the range between 0 and + π/4 in red (coded with “+1”). Phase difference values beyond these range were marked with green (coded with “0”) and represent non-synchronization. In the case of two persons, A and B, a blue stripe in the diagram indicates that the phase of person B precedes the phase of person A, whereas a red stripe indicates that the phase of person A precedes the phase of person B. We then counted the number of data points that were phase-locked separately in each of these two ranges. Before counting, successive points in the defined range (between - π/4 and + π/4) with a time interval shorter than the period of the corresponding oscillation at the given frequency (*T* = 1/*f*) were discarded from the analysis. As shown in Figure S1, this cleaning procedure effectively eliminated instances of accidental synchronization. Based on this count, we obtained two further synchronization indices in addition to the *PSI* measure: (1) the Absolute Coupling Index (*ACI*) and (2) the Integrative Coupling Index (*ICI*). *PSI* and *ACI* are symmetrical measures (i.e., 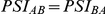 and 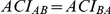) and have similar properties when synchronization is in phase. *ICI* is an asymmetrical measure (i.e., 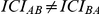), which emphasizes both the common (absolute) and the “positive” influence on phase synchronization (see Methods for details).

#### Granger Causality (GC)

We also calculated multivariate *GC* based on MultiVariate AutoRegressive (MVAR) modeling. *GC* is a directional coupling measure indicating the better predictability of a time series when using all available information (from other channels or participants) in contrast to considering the target time series alone (see Methods). This measure, which has become popular in the neurosciences [24], [25], [26], was first introduced by Wiener [27] and formalized by Granger [28] in the context of stochastic processes in econometrics.

#### Representation of synchronization patterns

**Figure 3:**
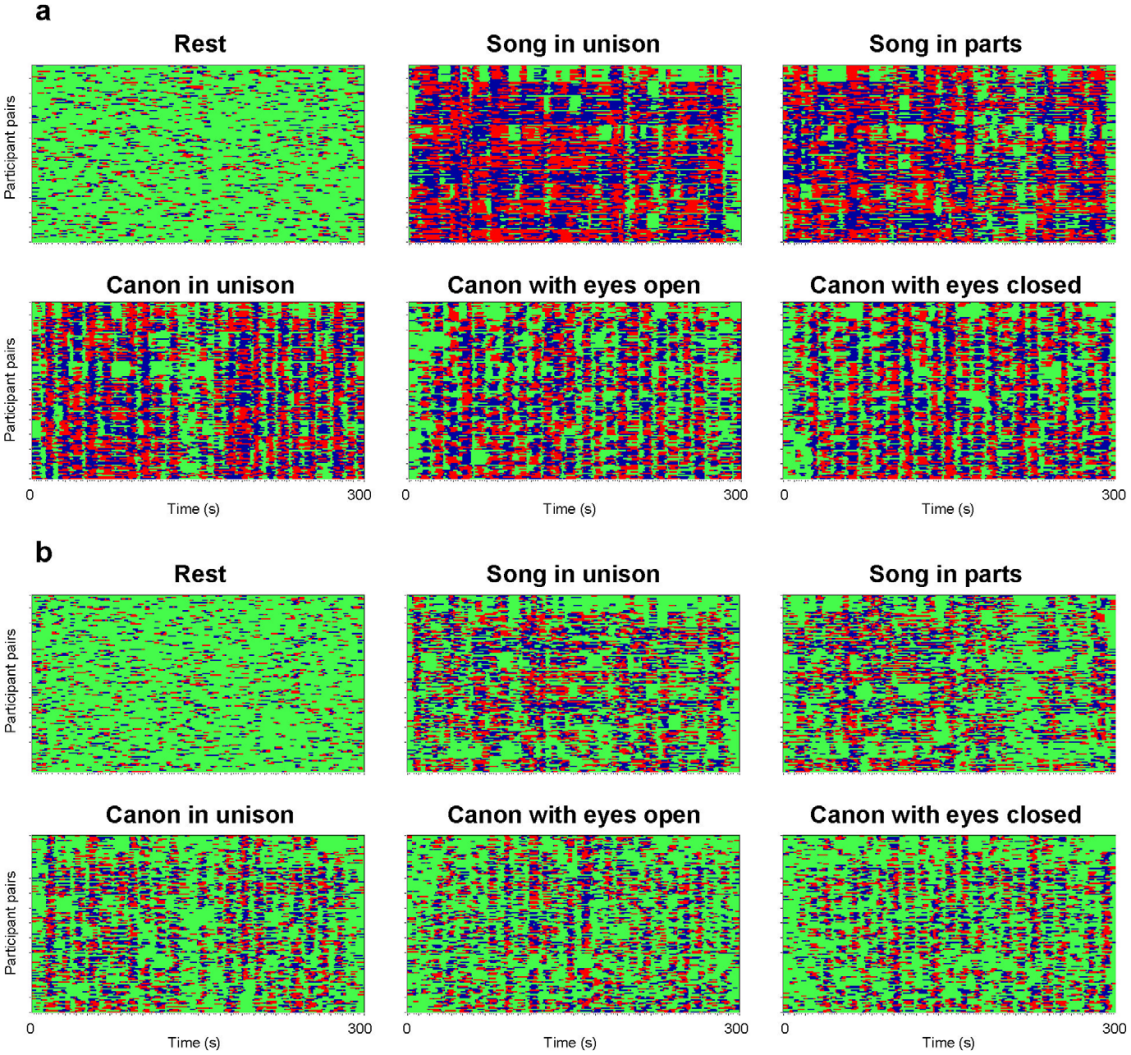
Phase synchronization patterns of the choir for respiration and HRV signals during rest and the five singing conditions. a, Phase synchronization patterns for respiration; b, Phase synchronization patterns for HRV. Each diagram contains 132 lines displaying synchronization pattern of all possible participant pairs in the choir. Color coding and structure of the diagram are in accordance with Fig. 2. These diagrams display the phase synchronization at the frequency of 0.24 Hz.

We calculated all indices for individual pairs. There were 132 pairs in the case of asymmetric coupling measures, and 66 in the case of symmetric measures. For representation and statistical analyses of these synchronization or coupling measures, we used the six frequencies (0.03, 0.05, 0.08, 0.11, 0.16 and 0.24 Hz) that showed prominent peaks in the PSD during singing. The time course of phase synchronizations for respiration and HRV between the choir participants (132 pairs) at the frequency of 0.24 Hz are shown in Figure 3, panels a and b, respectively. Corresponding data with synchronization patterns for the other five frequencies can be found in the Figures S2 and S3. The results showed that: (i) phase synchronization in general for both respiration and HRV was higher during singing than during rest, especially at higher frequencies; furthermore, synchronization patterns during singing were well structured across the participants, whereas those during the rest were mostly non-uniformly distributed; (ii) phase synchronization was generally higher when singing in unison than when singing with multiple voice parts; (iii) some participants tended to be early in their phase course (marked with red), whereas others tended to be late (marked with blue); for some participants, the phase courses were mixed; (iv) decoupling periods (marked with green) tended to be of short duration, at least for the respiration time series; the synchronization patterns at low frequencies (until 0.08 Hz) deviated from this pattern, especially for pairs singing different parts during canon and song. Decoupling patterns during four-part song singing were pronounced for the time period between 2.0 and 3.6 minutes, when the two male voices and the two female voices break up into two different melodic groups that are offset from each other and are apparently characterized by different respiration rhythms.

### Statistical analysis of coupling dynamics

Statistical analyses (two-way repeated measures ANOVA, Condition x Frequency) of the different coupling measures for both respiration and HRV showed strong Condition and Frequency effects (all Ps<0.0001) indicating increasing coupling with higher frequencies and stronger coupling during singing than during rest. Condition effects were more pronounced at higher frequencies, as indicated by a Condition-by-Frequency interaction (all Ps<0.0001). Post hoc one-way repeated measures ANOVAs carried out separately for different frequencies again revealed reliable condition effects (all Ps<0.001), above all due to the stronger synchronization during singing than during rest. The *GC* measure tested with one-way repeated measures ANOVA showed a strong condition effect indicating higher directed coupling during singing than during rest. Phase synchronization for both respiration and HRV was strongest for the song at the frequencies 0.11 and 0.24 Hz and for the canon at the frequencies 0.08 and 0.16 Hz. Irrespective of the frequencies, however, synchronization during unison singing was usually higher than during multiple-part/voice singing. There were some exceptions, including *PSI* at low frequencies and *GC*. Statistical details and corresponding graphical presentations of these effects can be found in Table 1 and 2, and in Figure S4.

**Table 1:** ANOVA results (F and P values) for the three coupling measures (*PSI*, *ACI*, and *ICI*) of respiration and HRV.

	Condition		Frequency		Condition x Frequency	
	F value	P value	F value	P value	F value	P value
	DF = 5,325		DF = 5,325		DF = 25,1625	
Respiration						
*PSI*	326.4	<0.0001 η^2^ = 0.83	55.2	<0.0001 η^2^ = 0.46	127.3	<0.0001 η^2^ = 0.66
*ACI*	129.0	<0.0001 η^2^ = 0.67	292.2	<0.0001 η^2^ = 0.82	84.7	<0.0001 η^2^ = 0.57
*ICI*	140.3	<0.0001 η^2^ = 0.52	248.1	<0.0001 η^2^ = 0.65	49.5	<0.0001 η^2^ = 0.27
HRV						
*PSI*	92.8	<0.0001 η^2^ = 0.59	48.3	<0.0001 η^2^ = 0.43	29.5	<0.0001 η^2^ = 0.31
*ACI*	35.6	<0.0001 η^2^ = 0.35	162.9	<0.0001 η^2^ = 0.72	22.0	<0.0001 η^2^ = 0.25
*ICI*	32.9	<0.0001 η^2^ = 0.20	241.1	<0.0001 η^2^ = 0.65	14.9	<0.0001 η^2^ = 0.10

**Table 2:** ANOVA results (F and P values) for the three coupling measures (*PSI*, *ACI*, and *ICI*) at different frequencies and *GC* of respiration and HRV.

Measures	Frequency	Condition (DF = 5,325)			Post-hoc *Tukey*-tests
		F value	P value	η^2^	
Respiration					
	0.05 Hz	83.0	<0.0001	0.56	A<C^*^<B<E<D^*^<F^*^
	0.08 Hz	200.0	<0.0001	0.76	A<C^*^<B^*^<E^*^<D^*^<F
	0.11 Hz	159.0	<0.0001	0.71	A<F^*^<E<C^*^<D<B^*^
	0.16 Hz	248.5	<0.0001	0.79	A<C^*^<B^*^<F<E<D (E<B^*^; F<B^*^)
	0.24 Hz	652.3	<0.0001	0.91	A<E^*^<F<D^*^<C^*^<B^*^
*ACI*	0.03 Hz	40.1	<0.0001	0.38	A<B<C<E<F<D^*^
	0.05 Hz	44.2	<0.0001	0.41	A<E<F<C^*^<B^*^<D^*^
	0.08 Hz	128.4	<0.0001	0.66	A<C^*^<B<E^*^<F<D^*^
	0.11 Hz	195.3	<0.0001	0.75	A<E^*^<F<D^*^<C^*^<B^*^
	0.16 Hz	229.2	<0.0001	0.78	A<C^*^<B^*^<E<F<D^*^
	0.24 Hz	381.7	<0.0001	0.85	A<E^*^<F<D^*^<C^*^<B^*^
*ICI*	0.03 Hz	43.9	<0.0001	0.25	F<E<A<B<C<D^*^
	0.05 Hz	64.6	<0.0001	0.33	E≤F≤A<C^*^<D<B
	0.08 Hz	90.6	<0.0001	0.41	A<B^*^<C<E^*^<F<D^*^
	0.11 Hz	109.4	<0.0001	0.46	A<F^*^<E<D^*^<C^*^<B
	0.16 Hz	194.4	<0.0001	0.60	A<C^*^<B^*^<E<F<D (E<D^*^)
	0.24 Hz	180.3	<0.0001	0.58	A<E^*^<F<D^*^<C<B (D<B^*^)
*GC*		30.7	<0.0001	0.19	A<C^*^<D<B<F<E (B<E^*^)
HRV					
*PSI*	0.03 Hz	16.9	<0.0001	0.21	C<A<B<E<D<F^*^ (C<B^*^; B<D^*^)
	0.05 Hz	10.9	<0.0001	0.14	A<D^*^<F<E<C<B
	0.08 Hz	18.5	<0.0001	0.22	A<C<B<E^*^<D<F
	0.11 Hz	39.4	<0.0001	0.38	A<F^*^<E<D<C^*^<B^*^
	0.16 Hz	78.4	<0.0001	0.55	A<C^*^<B^*^<E<D<F
	0.24 Hz	192.9	<0.0001	0.75	A<E^*^<F<D<C^*^<B^*^
*ACI*	0.03 Hz	3.8	<0.01	0.06	C<B<E<A<F<D (A<D^*^)
	0.05 Hz	10.9	<0.0001	0.14	A<F<E<D<B<C (A<E^*^; F<D^*^; E<C^*^)
	0.08 Hz	3.5	<0.01	0.05	F<E<B<A<C<D (F<C^*^)
	0.11 Hz	29.6	<0.0001	0.31	F<E<A<D^*^<C<B (D<B^*^)
	0.16 Hz	36.4	<0.0001	0.36	A<C^*^<B<E<F<D (C<E^*^; B<D^*^)
	0.24 Hz	103.4	<0.0001	0.61	A<F^*^<E<D<C^*^<B
*ICI*	0.03 Hz	3.1	<0.01	0.02	C<F<B<E<A<D (F<D^*^)
	0.05 Hz	11.0	<0.0001	0.08	F<A<E<D<B<C (A<D^*^;E<C^*^)
	0.08 Hz	4.9	<0.001	0.04	F<E<B<A<D<C (F<D^*^;E<C^*^)
	0.11 Hz	30.2	<0.0001	0.19	E<F<A<D^*^<C<B
	0.16 Hz	38.4	<0.0001	0.23	A<C^*^<B<E≤F<D (C<E^*^;B<D^*^)
	0.24 Hz	88.5	<0.0001	0.40	A<F^*^<D<E<C<B (F<C^*^; E<B^*^)
*GC*		15.0	<0.0001	0.10	A<C^*^<B<E<D<F (E<F^*^)

A =  rest; B =  singing song in unison; C =  singing a four-part song; D =  singing canon in unison; E =  singing a three-part canon with eyes open; F =  singing a three-part canon with eyes closed.

### Network connectivity structures

#### Degrees and strengths

The structure of network connectivity can be represented by undirected and directed graphs [29] (see Methods for documentation of connection thresholds). Degrees of a node provide information about the number of incoming (in-degrees) or outgoing (out-degrees) number of connections, and the strengths (also in- and out-strengths) reflect the overall strength of the nodes' in-flow and out-flow connections. The sum of the in- and out-degrees (strengths) contains information about the overall activity of a node, whereas the difference between out- and in-degrees (out-in) reflects the dominance of either the in- or the out-flow of the node's connections. Note that the symmetric measures (e.g., *PSI* and *ACI*) only contain information about the overall activity of the node, and do not differentiate between in-flow and out-flow.

**Figure 4:**
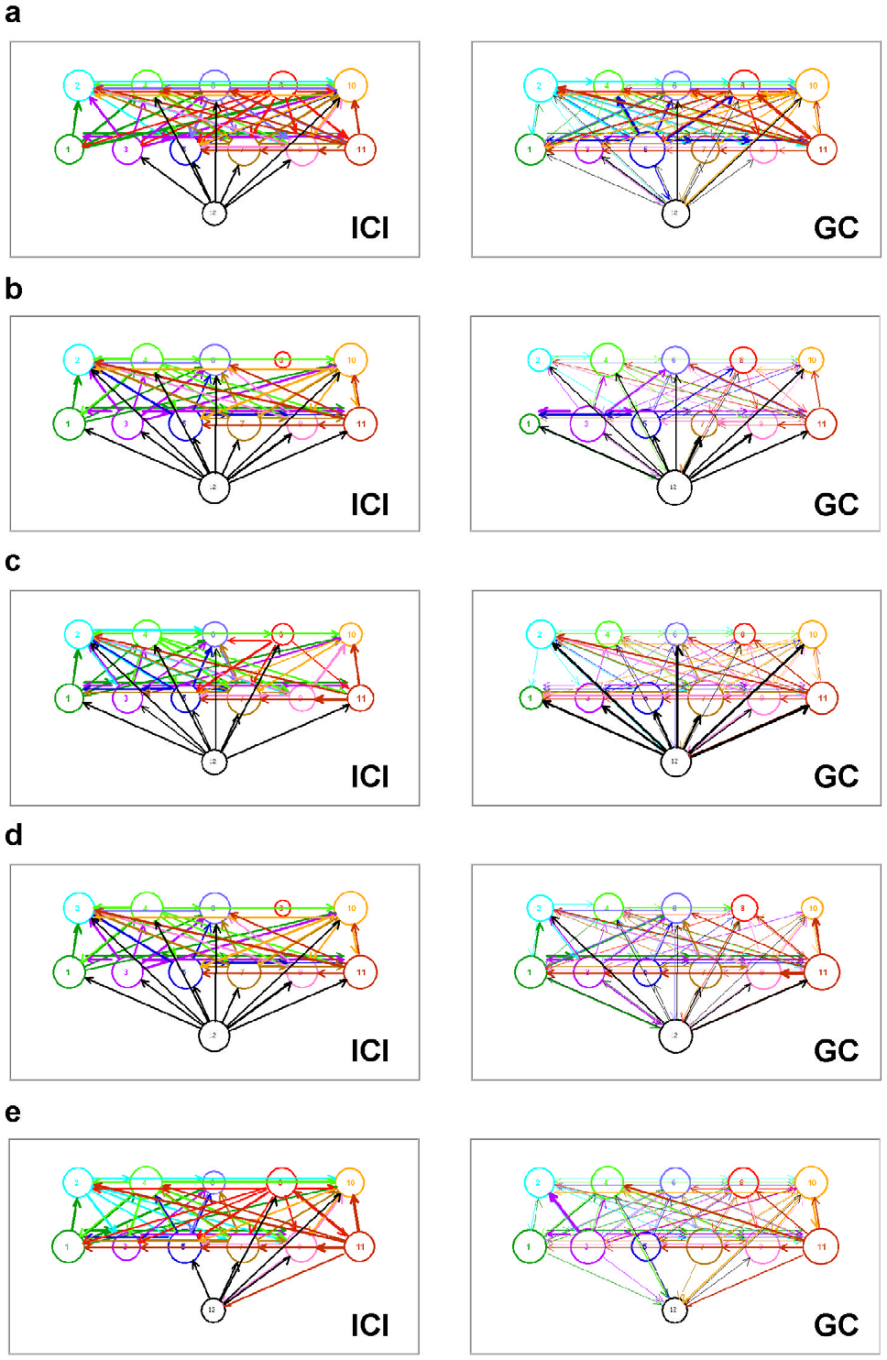
Directed connectivity networks for respiration *ICI* and *GC* measures during different singing conditions. **a**, song singing in unison. **b**, singing of the song in four parts. **c**, single canon entry sung in unison. **d**, canon singing with eyes open. **e**, canon singing with eyes closed. *ICI*-based networks are displayed on the left, and the *GC*-based networks are displayed on the right. The size of the circle representing choir participants depends on the number of both incoming and outgoing connections. The thickness of the links corresponds to the connection strength, and the arrow displays the direction of the causal influence. Note that the frequency of interest in the case of *ICI* corresponds to 0.24 Hz. (*ICI*  =  Integrative Coupling Index; *GC*  =  Granger Causality).

As shown in Figure 4 for respiration using *ICI* at the frequency of 0.24 Hz, the connections between the choir members were strongest while singing the song in unison (Figs. 4a) and also while singing the canon with eyes closed (Fig. 4e). The influence of the conductor was strongest during both the singing of the song in four parts (Fig. 4b) and the singing of the canon with eyes open (Fig. 4d; for related information on HRV, see Figure S5). Results obtained with *GC* confirm this result, with the difference that the influence of the conductor was strongest during singing of the single canon entry in unison (Fig. 4c). The connection of the conductor to the choir in these cases was predominantly directed from the conductor to the choir members. This indicates that changes in respiration patterns and, to some extent, changes in HRV (e.g., canon conditions) occurred earlier in the conductor than in the choir members, and were then transmitted from the conductor to the choir. This pattern is in accordance with the functional rule of the conductor in a choir. Further details about network connectivity and evidence on causal influences based on the *GC* measure are presented in detail in the Figure S6, S7, S8, and S9. The differences between out- and in-degrees (strengths) for directed measures, at least in the cases of *ICI* at high frequency (e.g., 0.24 Hz) and *GC*, again reflect the organizing role of the conductor.

#### Community structure, modularity, and Z-P parameter space

A common feature of complex systems or networks is their “community structure”, or the tendency of nodes to divide into groups or communities, with more internal than external connections [19], [20], [21]. By conceiving of participants as nodes in a network, one can derive network and node properties such as modularity (*M*), within-module degree (*Z_i_*) and participation coefficient (*P_i_*), among others. *M* reflects the overall property of the network to partition the nodes into modules; *Z_i_* indicates how well-connected a given node (i.e., participant) *i* is to other nodes in the module; *P_i_* describes how “well-distributed” connections of node *i* are among different modules.

#### Modularity structure during canon singing

**Figure 5:**
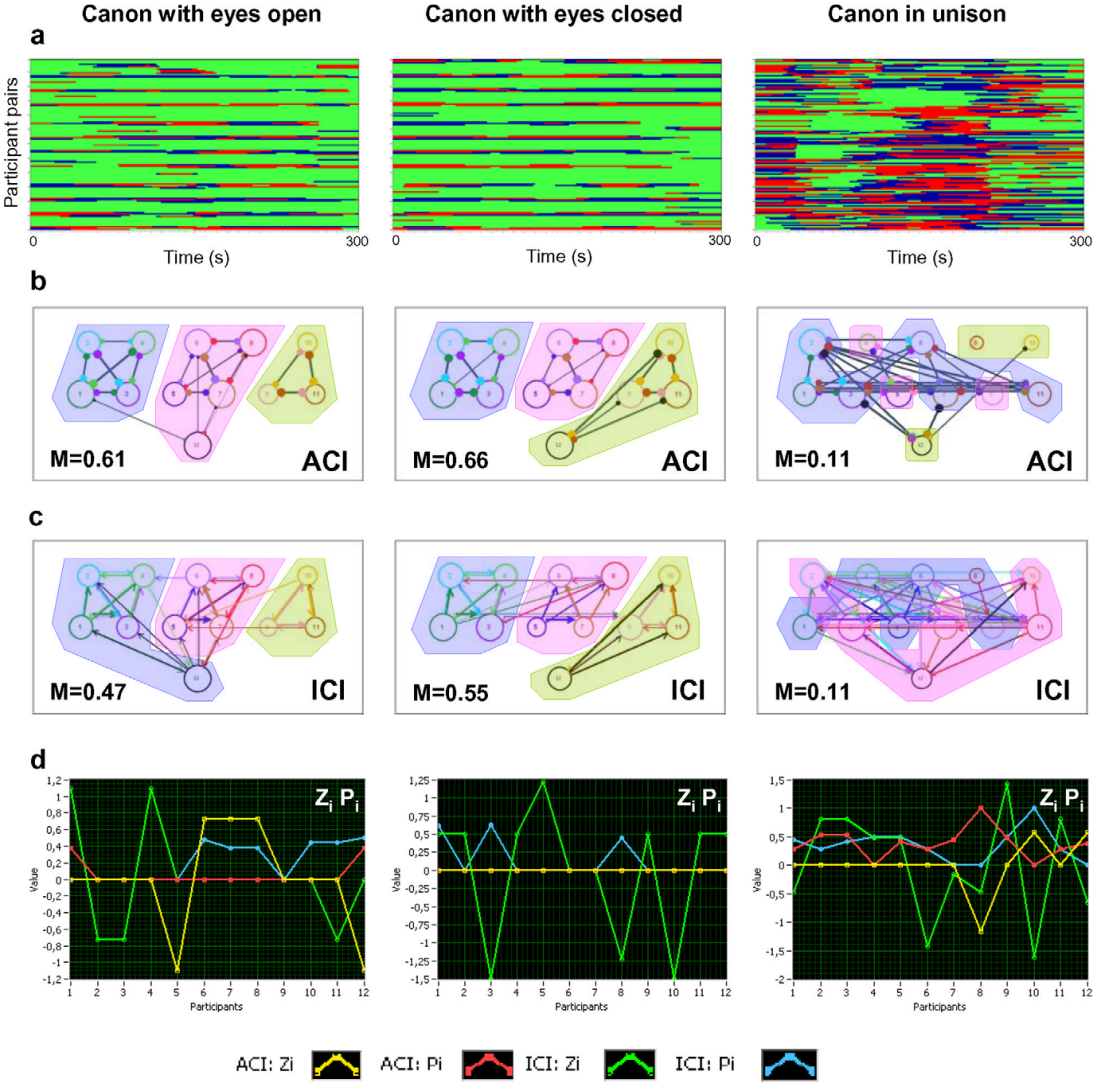
Phase synchronization patterns, connectivity networks, and modularity effects during the different canon singing conditions. a, Phase synchronization patterns for the three canon-singing conditions: canon singing with eyes open (left column), canon singing with eyes closed (middle column), and canon singing in unison (right column). b-c, The two rows of diagrams in the middle display undirected networks for *ACI* (b) and directed networks for *ICI* (c) measures. *ACI* and *ICI* were determined at the low frequency of 0.03 Hz. The colored areas display the partition of the networks into modules. d, diagrams reflecting the Z-P parameter space measures: within-module degree (*Z_i_*) and participation coefficient (*P_i_*); for more details, see legends at the bottom. Note that the modularity effect during canon singing in unison was low and the partition into modules is therefore blurred.

During canon singing, the directed (e.g., *ICI*) and undirected (e.g., *ACI*) coupling measures reflecting in-phase synchronization revealed a clear partitioning of the choir into different groups based on the parts sung in the choir. Interestingly, this partition effect was not observed during the singing in unison (see Figure 5). Specifically, *ACI* (Fig. 5b) and *ICI* (Fig. 5c) at low frequencies (i.e., at 0.03 and 0.05 Hz) showed partitioning of choir members during canon singing into the three different modules corresponding to the three canon parts. This partition was also accompanied by high values of modularity (*M*) during the singing of the canon with eyes closed (0.03 Hz: *M*_ACI_ = 0.66, *M*_ICI_ = 0.55; 0.05 Hz: *M*_ACI_ = 0.50, *M*_ICI_ = 0.33) and open (0.03 Hz: *M*_ACI_ = 0.61, *M*_ICI_ = 0.47; 0.05 Hz: *M*_ACI_ = 0.42, *M*_ICI_ = 0.28). However, *M*-values were very low or close to zero during the singing of the canon in unison. When the conductor sang along with the third entry during the singing of the canon with eyes closed, she was unambiguously assigned to this group. In the case of canon singing with eyes open, the conductor was partitioned into group 2 for the *ACI* measure (Fig. 5b) and into group 1 for the *ICI* measure (Fig. 5c). Because of the high connectedness within the modules and their high closeness, the within-module degree *Z_i_* and the partition coefficient *P_i_* were mostly zero (Fig. 5d). This was especially true for the *ACI* measure while singing the canon with eyes closed. Here the conductor could be strongly partitioned into the module with which she sang. While singing the canon with eyes open, the conductor was not so strongly connected within the module as the other singers and there are some variations of the *Z_i_* value in this module. In the case of the *ICI* measure, which reflects not only the overall synchronization level but also the earliness of the phase dynamics, there were more variations in *Z_i_* and *P_i_* values. This indicates the different roles of the choir members within and between the modules, although the partition into the modules is the same as in case of the *ACI* measure. In contrast to these two canon conditions, canon singing in unison showed relatively low modularity indices (0.03 Hz: *M*_ACI_ = 0.11, *M*_ICI_ = 0.11; 0.05 Hz: *M*_ACI_ = 0.07, *M*_ICI_ = 0.10) and more diffuse community structures (see for details Fig. 5b and 5c).

#### Modularity structure during song singing

**Figure 6:**
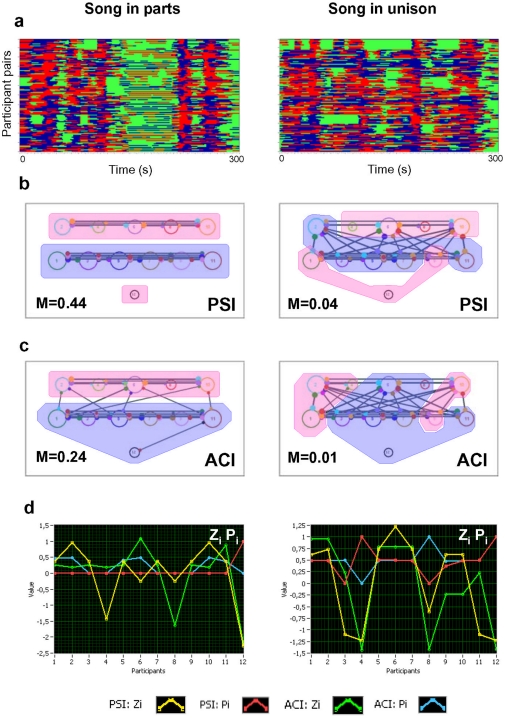
Phase synchronization patterns, connectivity networks, and modularity effects during the singing of the song in four parts and singing it in unison. a, Phase synchronization patterns for the two song-singing conditions: singing of the song in parts (left column), and song singing in unison (right column). b-c, The two rows of diagrams in the middle display undirected networks for *PSI* (b) and *ACI* (c) measures. *PSI* and *ACI* were determined at the moderate frequency of 0.11 Hz. The colored areas display the partition of the networks into modules. d, Diagrams reflecting the Z-P parameter-space measures: within-module degree (*Z_i_*) and participation coefficient (*P_i_*); for more details, see legends at the bottom. Note that the modularity effect during song singing in unison was low and the partition into modules is therefore blurred.

*PSI* failed to show the above mentioned strong modularity effects for canon singing, but showed strong modularity effects for the part song at several higher frequencies, especially at 0.11 Hz (see Figure 6), in the sense of separating female and male voices into two modules (*M*_PSI_ = 0.44, *M*_ACI_ = 0.24, *M*_ICI_ = 0.10). Interestingly, this separation into two modules was also seen at frequencies that did not show spectral peaks during the homophonic singing of the song (0.08 and 0.16 Hz). Furthermore, the participation coefficient *P_i_* was zero (e.g., for *PSI*) or close to zero (e.g., for *ACI*) indicating the within-module homogeneity of the two modules. Also, the within-module degree *Z_i_* in all females was at least 0.38 in the case of *PSI* and 0.26 in the case of *ACI* (with the exception of subject 11, with *Z_i_* = 0.88), indicating strong connectedness among female choir members. The male participants were less connected to each other and showed different *Z_i_* values (see Fig. 6d for details) indicating variable connectivity within the module. Like the canon condition, song singing in unison showed relatively low modularity indices (*M*_PSI_ = 0.04, *M*_ACI_ = 0.01, *M*_ICI_ = 0.10) and more diffuse community structures (see Fig. 6b and c for details).

To test whether the partitioning into modules was in fact related to singing different parts, we calculated corresponding synchronization and modularity measures separately for the three consecutive 100-s time intervals. It can be seen (Fig. S10) that the above-mentioned modularity structure was strongly related to the time interval where the split between the male and female voices occurred (*PSI*: *M*_1_ = 0.0, *M*_2_ = 0.48, *M*_3_ = 0.12; *ACI*: *M*_1_ = 0.0, *M*_2_ = 0.49, *M*_3_ = 0.15; *ICI*: *M*_1_ = 0.07, *M*_2_ = 0.23, *M*_3_ = 0.11). Moreover, the modularity effect after the split occurred was considerably stronger than in the whole singing interval.

## Discussion

We examined synchronization of respiration and HRV among choir members and their conductor singing a song or a canon under different conditions relative to rest. The main findings are that: (a) singing conditions showed several prominent PSD peaks for both respiration and HRV indicating RSA changes during singing; (b) phase synchronization between choir participants for both respiration and HRV was generally higher during singing than during rest, especially at higher frequencies; furthermore, synchronization indices were generally higher during singing in unison than when singing with multiple voice parts; (c) directed measures (*GC* and *ICI* at the high frequency) showed strong, mostly unidirectional influences of the conductor on the choir members indicating that changes in respiration and HRV occurred earlier in the conductor than in the choir participants, in accordance with the conductor's functional role; (d) the choir members singing different parts of song or canon could be partitioned into modules using graph-theoretical measures applied to directed and undirected synchronization indices.

### Spectral power peaks

Spectral power peaks found in low- and high-frequency ranges indicated activation of both sympathetic and parasympathetic nervous systems during singing. This finding is in line with the results of Grape et al. [17] for professional singers. Note that the choir members were standing both while singing and while being at rest. Apparently, then, singing induces high-frequency changes in respiration and HRV despite the commonly observed suppression of high-frequency fluctuations while standing [14]. In our study, spectral peaks discriminated not only between rest and singing conditions but varied by singing activity.

### Synchronization patterns and evidence for causal influences

Though respiration synchronization among choir members during singing was more pronounced than HRV synchronization, comparisons to rest indicate that increases in synchronization were present for both sets of measures. We observed stronger synchronization at high frequencies (> 0.15 Hz) during singing compared to rest, perhaps reflecting, at least in part, their greater spectral power. However, in some cases, large effects were also found for low frequencies, especially at 0.08 Hz, which is a peak frequency for the rest condition. Synchronization patterns during singing followed the different parts of the music, whereas those during rest were mostly non-uniformly distributed. Some participant pairs during canon singing were not synchronized at low frequencies (e.g., 0.03 and 0.05 Hz) due to their singing different parts. This decoupling in some pairs reduced the overall degree of synchronization, at least for in-phase synchronization measures (e.g., *ACI* and *ICI*).

Synchronization was greater during unison singing (both song and canon) than during four-part and canon singing. The differences in synchronization between canon singing with open and closed eyes (or with and without the conductor's influence) were quite small and often not statistically reliable. Interestingly, we found clear evidence in favor of a causal influence of the conductor. This influence was directed from the conductor to the choir members during canon singing with eyes open, and partially reversed with eyes closed. Apparently, the conductor tried to follow the choir when lack of visual input prevented her from taking the lead. Evidence for a strong causal influence of the conductor on the choir was also found during singing of the song in four parts. Taken together, these results highlight the organizing role of the conductor in the choir. It appears that the conductor also influenced the choir members' HRV. Also, the influence of the conductor on choir members during the singing of the canon entry in unison was larger than during normal canon singing with eyes open, at least for the *GC* measure. When singing in unison, the conductor may have distributed her attention equally across the entire choir during the whole period of singing. However, during canon singing, the conductor may have switched her attention and, accordingly, her respiration patterns, to influence specific parts of the choir more than others. Relative to unison singing, this switching process during canon singing may have attenuated the conductor's influence as captured in the present analyses.

### Modularity effects during choir singing

Modularity analyses are often applied to larger and more complex networks (cf., [19], [20], [21], [30]) than the present system of eleven choir members and a conductor. Our results show that such analyses are applicable to smaller networks such as a choir or other structured multi-person interactions. As expected, we found modularity effects during singing of the song in four voice parts and during the singing of the canon with three entries, both with eyes open and closed. It should be noted that these modularity effects could not be found in the unison conditions of both song and canon. Modularity effects were present at low frequencies (e.g., 0.03 and 0.05 Hz) in the canon condition and at moderate frequency frequencies (e.g., 0.08–0.16 Hz) in the song condition. We can offer two potential reasons why the modularity was found at low frequencies during canon singing: (i) the frequency of 0.027 Hz is the basic frequency of the six harmonics that show spectral peaks during canon singing; (ii) the time shift between the canon parts is about 12.5 s and corresponds approximately to the one third of the period of the basic frequency (the frequency where the modularity effect was found). During the singing of the song in four parts, the frequency of 0.11 Hz showed the most prominent PSD peak. It is therefore not surprising that the strongest modularity effect was found at and around this frequency (e.g., 0.08 and 0.16 Hz).

The *PSI* failed to show strong modularity effects for canon singing, but showed strong modularity effects for the homophonic song at some higher frequencies (e.g., 0.08–0.16 Hz) separating female and male voices into the two modules. Most intriguing is the fact that the *PSI* measure normally reflects not only in-phase synchronization but is also oriented on the phase differences, regardless of their phase angles (i.e., in the case of a high *PSI* value, the phase difference could be constant across time but not obviously zero). In the canon conditions, the choir members sang the same text shifted in time, so the phase differences in pairs belonging to different groups were not equal but constant through time within the pair and showed, accordingly, high *PSI* values regardless of module membership. On the other hand, other coupling measures (e.g., *ACI* and *ICI*) showed high in-phase synchronization within the modules but zero synchronization between the modules (see Fig. 5a and b for details). In the case of homophonic singing of the song, the texts sung by female and male voices differed and so did their respiration rhythms, at least in the interval between 2.0 and 3.6 minutes. This is probably the reason why female and male singers formed two separate modules, also detected by the *PSI* measure, and why phase synchronization was high within, but low between the two modules.

The strongest evidence supporting the hypothesis of a causal effect of the conductor on the choir members was found at high frequencies that presumably represent very fast modulations of the respiration rhythm. These modulations are likely to represent continuous interventions of the conductor that are aimed at stabilizing the choir's singing pace. The results obtained with the *GC* measure are particularly clear in this regard because they emphasize the influence of the “past” conductor's respiration pattern on the “present” respiration rhythm of the choir members. Our results indicate that high-frequency modulations in respiration and HRV present study embody the leader-follower relation between the conductor and the choir members during choral singing.

Methodologically, this study shows that data acquisition and analysis methods for simultaneous respiration and ECG recordings from multiple persons are instrumental in discovering oscillatory couplings during interpersonal interactions. We have demonstrated how such joint dynamics or networks can be investigated. We have also shown that the modulation of different synchronization measures, determined at different frequencies in respiration and HRV, can be attributed to different roles in social interaction. In this sense, singing in a choir served as a model for a large number of communicative settings. Future research needs to explore interpersonal oscillatory couplings as a general mechanism supporting communication, voluntary action coordination, and development.

## Methods

### Participants

Five men and seven women (age range  = 23.06–56.68; M = 35.7; SD = 10.69) were recruited from the choir of the Max Planck Institute for Human Development in Berlin, Germany. The group consisted of a conductor and eleven singers. The choir had maintained a regular, 2-hour-long weekly rehearsal schedule. The mean length of singing training among the subjects was 11.67 years (SD = 7.5) with a mean singing duration of 4.25 hours per week (SD = 3.35). Two of the twelve participants had professional musical training. Except for one person, all participants were able to play at least one instrument. The study was approved by the ethics committee of Max Planck Institute for Human Development (Berlin), and performed in accordance with the ethical standards laid down in the 1964 Declaration of Helsinki. All subjects volunteered for this experiment and gave their written informed consent prior to their inclusion in the study.

### Procedure

After providing written informed consent and completing a questionnaire related to musical experience and demographic information, the participants were connected to the measuring instruments. The experiment was conducted in a quiet room. The participants were aligned in a predetermined position with the eleven singers facing the conductor and standing in two rows. The testing session started and ended with a relaxation condition. The part song “Sally Gardens” in D major (Irish Folksong) and the canon “Signor Abbate” in B major (by Ludwig van Beethoven) were performed in several experimental variations (see Methods S1 for details). Both the song and the canon had been newly included in the choir's repertoire, and received some initial practice. All recordings were obtained with the subjects in a standing position and lasted five minutes per condition. The tasks were separated by three-minute breaks, and the song and canon tasks were divided by a longer ten-minute break during which the participants had the opportunity to sit down. The spatial positions of the choir members and the conductor, the order of experimental conditions, and a video recording of the singing, together with the physiological measures, can be found in Methods S1 and Video S1.

### Data acquisition and analysis

Electrocardiogram (ECG), respiratory movement, and vocal audio signals were recorded simultaneously for all participants during each of the twelve conditions. The surface ECG was obtained using small-surface Ag/AgCl monitoring electrodes (Ambu Blue sensor P). The first electrode was placed on the upper part of the sternum over the first rib between the two collarbones. The second electrode was attached under the last rib on the left lateral side of the medium range of the abdomen and the ground electrode was placed next to the second electrode arranged more medially on the abdomen. Before attaching the electrodes, the skin was cleaned with alcohol to keep the resistance low. Respiratory movements were measured using an elastic respiratory belt, which was placed around each participant's chest just below the axilla for transduction of rib cage circumference. Vocal signals were acquired with a head-set condenser microphone (Mc Crypt). The holders were placed behind the participantś ears and the spherical microphone was positioned roughly 1–2 cm from the participantś mouths. In addition, two 1D acceleration sensors were applied on the conductor's hands to register her hand movements. All signals from all participants were sampled simultaneously with a 5000 Hz sampling rate using BrainAmp ExG bipolar amplifiers and BrainVision Recorder software (Brain Products GmbH, Gilching, Germany). The antialiasing filter was set to 1000 Hz. Simultaneously with the electrophysiological measures, video was recorded with BrainVision Video Recorder software.

The QRS complexes in the ECG signals were used to identify beat locations. Once the timing of beats was determined, an instantaneous Heart Rate (HR) signal was created. Thereafter, HR and respiration signals were down-sampled to 4 Hz. The Spencer's 15-Point Moving Average method was used to smooth a time series in order to highlight the underlying structure. Furthermore, mean and trends were removed from the HR and respiration data, and then the data were normalized to a unit variance.

In the HR and respiration data, power spectral density was calculated using Fast Fourier Transform (FFT) with Hanning to determine spectral peaks during the rest and the singing conditions.

#### Calculation of synchronization indices

To investigate phase synchronization, we applied an analytic or complex-valued Morlet wavelet transform to compute the instantaneous phase in the frequency range from 0 to 2 Hz in 0.002-Hz steps. The complex mother Morlet wavelet, also called Gabor wavelet, has a Gaussian shape around its central frequency *f*: 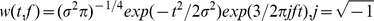
where σ is the standard deviation of the Gaussian envelope of the mother wavelet. The wavelet coefficients were calculated with a time step of 4 leading to time resolution of 1 sec.

In order to identify the phase relations between any two subjects/channels during the task, the instantaneous phase difference Δϕ*_mn_*(*t*, *f*) was computed from the wavelet coefficients for all possible subject pairs. Two different synchronization measures were obtained from these phase differences for the frequency of interest *f_i_*. Firstly, we obtained the Phase Synchronization Index (*PSI*), which is defined by 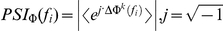, where 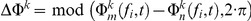 is the phase difference with instantaneous phases of these two signals across k data points during the task condition; 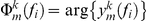 and 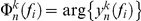. The *PSI* measure is in this sense similar to phase coherence, with the difference that *PSI* measures phase stability or phase invariance across the time within a trial or time series (e.g., [31]).

Given the estimates of the phase difference between pairs of signals (participants), it is then possible to ascertain how long the phase difference remains stable in defined phase angle boundaries by counting the number of points that are phase-locked in a defined time window. We adapted and slightly modified the procedure described in Kitzbichler et al. [23] by dividing the range between -1/4π and +1/4π into two ranges and distinguishing between positive and negative deviations from phase zero. As depicted in Figure 2, we marked negative deviations in the range between -π/4 and 0 in blue (coded with “-1”) and the positive deviations in the range between 0 and +π/4 in red (coded with “+1”). Phase difference values beyond these ranges were marked in green (coded with “0”) and represent non-synchronization. In the case of two persons, A and B, a blue stripe in the diagram would mean that the phase of person B precedes the phase of person A and a red stripe would mean that the phase of person A precedes the phase of person B. We then counted the number of data points that are phase-locked separately in each of these two ranges. Before counting, successive points in the defined range (between -π/4 and +π/4) with a time interval shorter than the period of the corresponding oscillation at the given frequency (*T* = 1/*f*) were discarded from the analysis. This cleaning procedure effectively eliminated instances of accidental synchronization. On the basis of this counting, we obtained different synchronization indices: (1) Positive Coupling Index, *PCI* =  relative number of phase-locked points lying in the positive range (between 0 and +1/4π); (2) Negative Coupling Index, *NCI* =  relative number of phase-locked points lying in the negative range (between -1/4πand 0); (3) Absolute Coupling Index, *ACI* =  relative number of phase-locked points lying in the whole range (between -1/4π and +1/4π) indicating in-phase synchronization (e.g., [23]); (4) Integrative Coupling Index, 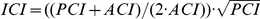. It could be seen that the *ICI* equals 1 when all points are phase-locked and lie in the positive range; when all phase-locked points lie in the negative range, the term 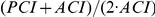 will approach 0.5, but through multiplication with 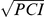 it will approach 0. In this way, the *ICI* measure ranges between 0 and 1 and is asymmetrical (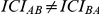), indicating both the common (absolute) and the “positive” influence in phase synchronization. All previous coupling measures are related to the whole number of points in the window and also range between 0 and 1. *PSI* and *ACI* are symmetrical measures (i.e., 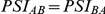 and 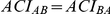) and have similar properties when synchronization is in phase. Positive and Negative Coupling Indexes are asymmetrical in the sense that 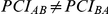, but they are symmetrical in the sense that 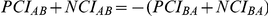 or 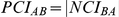.

#### Calculation of Granger Causality

In addition to the *ICI* measure described above, we used Granger Causality (*GC*) based on MultiVariate AutoRegressive (MVAR) modeling to investigate the directionality of coupling. In its original formulation *GC* is a bivariate concept but it can be extended to become multivariate. Given two time series *x*_1_ and *x*_2_, the model considered by Granger [28] assumes that x_1_(t) and x_2_(t), with t  =  1, … , T, are represented by a univariate AR model of order *p* as: 
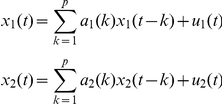
where the prediction error for a time series depends only on its own past, and by a bivariate ARX model as:
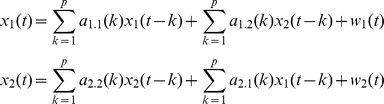
where the prediction error depends on the past of both signals. This model can also be extended (e.g., [25]) to Q signals x_1_, x_2_, … , x_Q_:
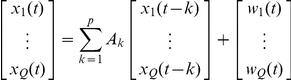
with
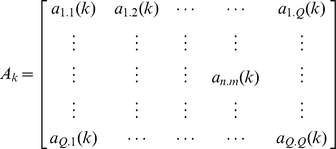

*A_k_* is the matrix of autoregressive coefficients for the *k*th time lag evaluating linear interaction of *x_m_(t-k)* on *x_n_(t)*, while *p* is the model order that gives the maximum number of time lags.

In the case of the bivariate GC, the unbiased variance of prediction error 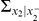 for the univariate AR model is given by: 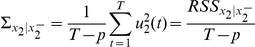
and the unbiased variance of prediction error 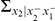 for the bivariate ARX model can be determined as:
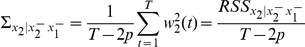
where 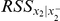 and 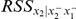 are the residual sums of squares in the corresponding models.

If X_1_ causes X_2_ in the Granger sense, then 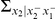 must be smaller than 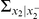. The level of linear causality is then estimated by
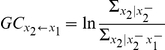
.

In the multivariate case, the causality from *x*_m_ to *x*_n_ could be obtained analogously as
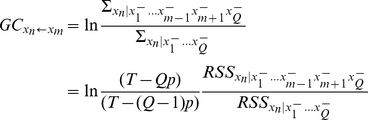
where 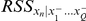 is the residual sum of squares for a variable *x*_n_ in the model involving all variables, and 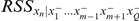 is the one for the same model involving all variables except *x*_m_.

#### Determination of network properties

To determine the network properties by means of the graph-theoretical approach, the threshold of the synchronization or coupling measures should be calculated. For this purpose, we first calculated coupling indices separately for the six different frequencies and conditions combined in four groups: (i) song singing in unison, (ii) song singing in parts, (iii) canon singing in unison, and (iv) canon singing in parts (with eyes open and closed). All repetitions of the corresponding conditions were entered into calculation. Next, we used a bootstrapping procedure by constructing 1000 resamples of the observed datasets of each of the coupling measures. The threshold was then determined as a mean plus the confidence interval at the significance level of 0.0001. Only the coupling values lying above the threshold were considered as a link or edge in the given network.

In the case of undirected measures (e.g., *PSI* and *ACI*), we determined the degree of a node *i* as the number of links connected to a node: 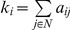, where 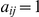 when link (*i,j*) exists, and 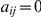, otherwise (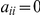 for all *i*). For directed measures (e.g., *ICI* and *GC*), we obtained the degree as a sum of in-degree and out-degree, where in-degree is a sum of all incoming connections 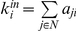, and out-degree is a sum of all outgoing connections 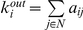. In the case of strengths for all of the measures, the sum of links would be replaced by the sum of weights, 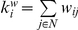. Thus, the strength could be considered as a weighted degree.

Community structures for weighted undirected und directed networks as well as indices of modularity (*M*), within-module degree (*Z_i_*), and participation coefficient (*P_i_*) were determined using Brain Connectivity Toolbox [29] (http://sites.google.com/a/brain-connectivity-toolbox.net/bct/Home). The modularity measure *M* describes how well a network may be delineated into communities or modules [20], [21] and is given for a weighted network by
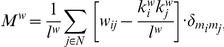
where 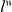 is the total number of edges in the network, N is the total number of nodes in the network, 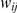 are connection weights, 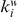 and 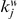 are weighted degrees or strengths of the nodes, and 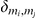 is the Kronecker delta, where 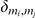 =  1 if *m_i_*  =  *m_j_*, and 0 otherwise. High modularity values indicate strong separation of the nodes into modules. M  =  0 if nodes are placed at random into modules or if all nodes are in the same cluster [19].

The within-module degree *Z_i_* indicates how well-connected node *i* is to other nodes in the module and is determined by
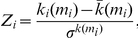
where *m_i_* is the module containing the node *i*, *k_i_*(*m_i_*) is the within-module degree of *i* (the number of links between *i* and all other nodes in *m_i_*), 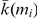 and 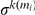 are the respective mean and standard deviation of the within-module *m_i_* degree distribution. Note that the within-module degree *Z_i_* is 0 if all of the nodes within the module have the same number of edges (e.g., if all the nodes within the module are fully interconnected with each other), otherwise it could have negative or positive values dependent on the number of links at different nodes.

The participation coefficient *P_i_* describes how well the nodal connections are distributed across different modules:
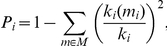
where M is the set of modules, *k_i_(m_i_)* is the number of links between node *i* and all other nodes in module *m_i_*, and, *k_i_* is the total degree of node *i* in the network. *P_i_* of a node *i* is therefore close to 1 if its links are uniformly distributed among all of the modules and 0 if all its links are within its own module. *Z_i_*- and *P_i_*-values are characteristic for the different roles of the nodes in the network [19].

### Statistical analysis

The three coupling measures (i.e., *PSI*, *ACI*, and *ICI*), which were determined at six different frequencies, were applied to a two-way repeated measures ANOVA with the two within-subject factors, Condition and Frequency. Thereafter, a one-way repeated measures ANOVA with the within-subject factor Condition was applied separately for each frequency and also for the *GC* measure followed by a post-hoc Tukey test. In all ANOVAs, Greenhouse-Geisser epsilons were used for non-sphericity correction when necessary. Before statistical testing, *GC* was normalized by the natural logarithmic transformation.

## Supporting Information

Figure S1 **Cleaning procedure to eliminate accidental synchronization points. a,** Synchronization pattern before cleaning. **b**, Synchronization pattern after cleaning. Note that during this cleaning procedure, successive points in the defined range (between -π/4 and +π/4) with a time interval shorter than the period of the corresponding oscillation at the given frequency (*T* = 1/*f*) were discarded.
(TIF)

Figure S2 **Synchronization patterns of respiration under the different task conditions for the five frequencies of interest.** **A - E**, Frequencies of interest: 0.03, 0.05, 0.08, 0.11, and 0.16 Hz, respectively. **a**, song singing in unison. **b**, singing of the song in four parts. **c**, single canon entry sung in unison. **d**, canon singing with eyes open. **e**, canon singing with eyes closed. Each diagram contains 132 lines displaying synchronization pattern of all possible participant pairs in the choir. The phase differences (Δϕ) were color-coded: blue stripes when - π/4 <Δϕ<0; red stripes when 0<Δϕ<+ π/4; and green stripes  =  non-synchronization when Δϕ< - π/4 or Δϕ> + π/4.
(TIF)

Figure S3 **Synchronization patterns of HRV under the different task conditions for the five frequencies of interest.** **A - E**, Frequencies of interest: 0.03, 0.05, 0.08, 0.11, and 0.16 Hz. a, Rest condition; b, Song singing in unison; c, Song with choral singing; d, Canon singing in unison; e, Choral singing of the canon with eyes open; f, Choral singing of the canon with eyes closed. Each diagram contains 132 lines displaying synchronization patterns of all possible participant pairs in the choir. The phase differences (Δϕ) were color-coded: blue stripes when - π/4 <Δϕ<0; red stripes when 0 <Δϕ< + π/4; and green stripes  =  non-synchronization when Δϕ< - π/4 or Δϕ> + π/4.
(TIF)

Figure S4 **Graphic representation of statistical results.** Means and standard error bars of the four synchronization measures (*PSI*, *ACI*, *ICI*, and *GC*) are displayed for respiration (left column) and HRV (right column) under the six different task conditions (Rest, S_uni, S_cho, C_uni, C_eo, and C_ec) and for the six frequencies of interest (0.03, 0.05, 0.08, 0.11, 0.16, and 0.24 Hz). Note that *GC* is frequency-independent and was normalized using natural logarithmic transform. (S_uni  =  singing song in unison; S_cho  =  singing a four-part song; C_uni  =  singing canon in unison; C_eo  =  singing a three-part canon with eyes open; C_ec  =  singing a three-part canon with eyes closed)
(TIF)

Figure S5 **Directed connectivity networks for HRV** ***ICI*** **and** ***GC*** **measures during the different singing conditions. a**, singing song in unison. **b**, singing a four-part song. **c**, singing canon in unison. **d**, singing a three-part canon with eyes open. **e**, singing a three-part canon with eyes closed. *ICI*-based networks are displayed at the left, and the *GC*-based networks are displayed at the right. The size of the circle representing choir participants depends on the number of all incoming and outgoing connections. The thickness of the links corresponds to the connection strength, and the arrow displays the direction of the causal influence. Note that the frequency of interest in the case of *ICI* corresponds to 0.24 Hz and *GC* is frequency-independent.
(TIF)

Figure S6 **Out- and In-degrees as well as the difference between them for respiration** ***ICI*** **and** ***GC*** **measures during the different singing conditions. a**, degrees for *ICI* measure during song singing. **b**, degrees for *GC* measure during song singing. **c**, degrees for *ICI* measure during canon singing. **d**, degrees for *GC* measure during canon singing. (S_uni  =  singing song in unison; S_cho  =  singing a four-part song; C_uni  =  singing canon in unison; C_eo  =  singing a three-part canon with eyes open; C_ec  =  singing a three-part canon with eyes closed)
(TIF)

Figure S7 **Out- and In-strengths as well as the difference between them for respiration** ***ICI*** **and** ***GC*** **measures during the different singing conditions. a**, strengths for *ICI* measure during song singing. **b**, strengths for *GC* measure during song singing. **c**, strengths for *ICI* measure during canon singing. **d**, strengths for *GC* measure during canon singing. (S_uni  =  singing song in unison; S_cho  =  singing a four-part song; C_uni  =  singing canon in unison; C_eo  =  singing a three-part canon with eyes open; C_ec  =  singing a three-part canon with eyes closed)
(TIF)

Figure S8 **Out- and in-degrees as well as the difference between them for HRV** ***ICI*** **and** ***GC*** **measures during the different singing conditions. a**, degrees for *ICI* measure during song singing. **b**, degrees for *GC* measure during song singing. **c**, degrees for *ICI* measure during canon singing. **d**, degrees for *GC* measure during canon singing. (S_uni  =  singing song in unison; S_cho  =  singing a four-part song; C_uni  =  singing canon in unison; C_eo  =  singing a three-part canon with eyes open; C_ec  =  singing a three-part canon with eyes closed)
(TIF)

Figure S9 **Out- and in-strengths as well as the difference between them for HRV** ***ICI*** **and** ***GC*** **measures during the different singing conditions. a**, strengths for *ICI* measure during song singing. **b**, strengths for *GC* measure during song singing. **c**, strengths for *ICI* measure during canon singing. **d**, strengths for *GC* measure during canon singing. (S_uni  =  singing song in unison; S_cho  =  singing a four part song; C_uni  =  singing canon in unison; C_eo  =  singing a three-part canon with eyes open; C_ec  =  singing a three-part canon with eyes closed)
(TIF)

Figure S10 **Connectivity networks and modularity effects during singing of the song in the three 100-s consecutive time intervals.** *PSI* and *ACI* were determined at the moderate frequency of 0.11 Hz. The colored areas display the partition of the networks into modules. **a**, the first 100-s time interval. The modularity M is zero, and the choir could not be divided in any modules. **b**, the second 100-s time interval. The choir is strongly divided into two modules separating female and male voices. **c**, the third 100-s time interval. The modularity M has moderate values, and the female and male voices are mixed in the two modules.
(TIF)

Methods S1 **Experimental design and procedure with position of the choir participants and the conductor in the choir during singing as well as the order of test conditions.**
(DOC)

Video S1 **Position of the choir participants and the conductor in the choir during singing.** This movie shows the simultaneous video and electrophysiological recordings of the choir while singing a four-part song (“Sally Gardens” in D major, Irish Folksong). ECG (ECG01-ECG12) and respiration (ATM01-ATM12) of a conductor and eleven singers as well as the hand movements of the conductor (ACT01 and ACT02).
(MP4)
