# Supplementary figures and images for: Cardiac and Respiratory Patterns Synchronize between Persons during Choir Singing
Source: PLoS One. 2011 Sep 21;6(9):e24893. doi: 10.1371/journal.pone.0024893 (PMC3177845; doi:10.1371/journal.pone.0024893)

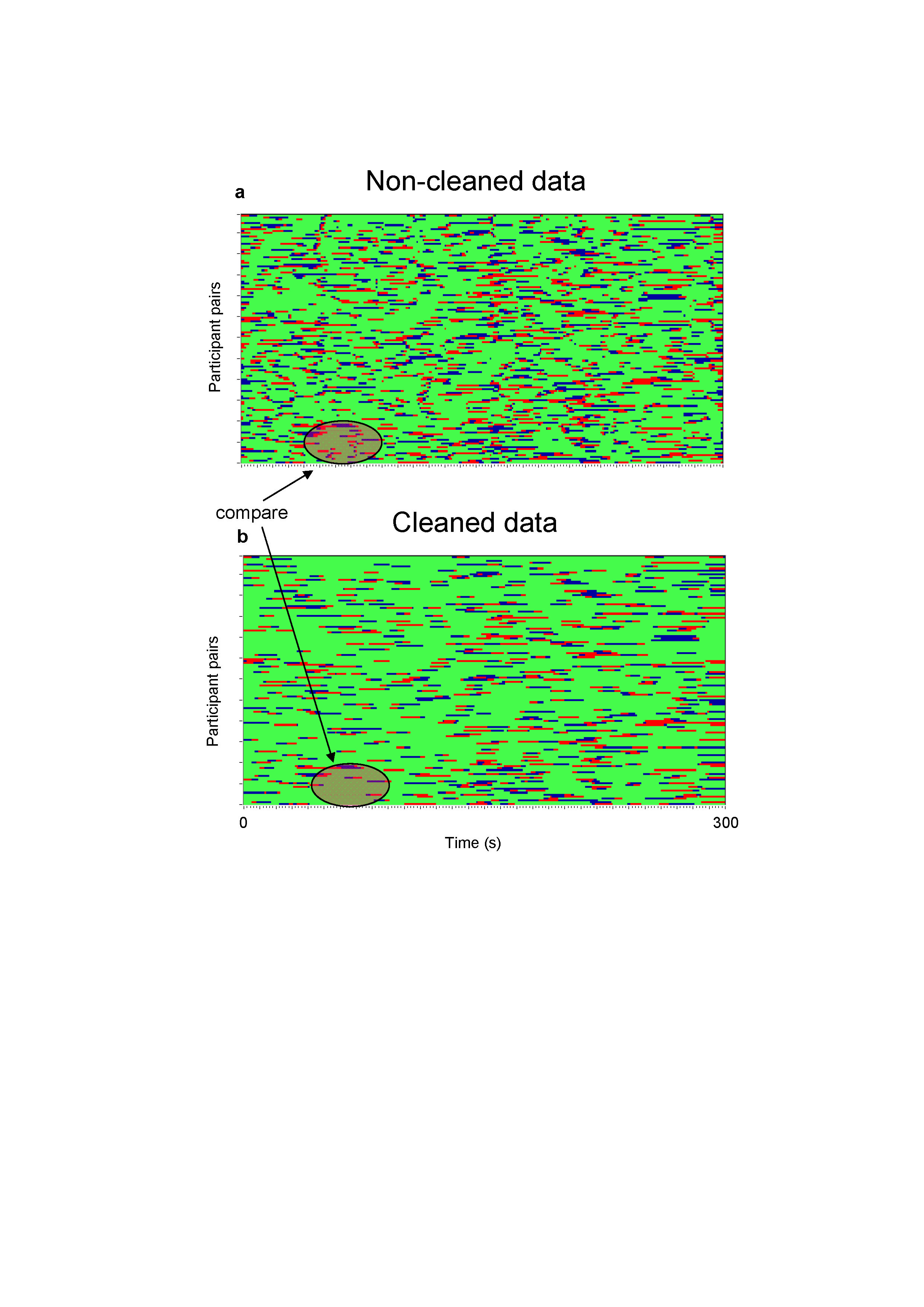

Supplement: Figure S1 — Cleaning procedure to eliminate accidental synchronization points. a, Synchronization pattern before cleaning. b, Synchronization pattern after cleaning. Note that during this cleaning procedure, successive points in the defined range (between -π/4 and +π/4) with a time interval shorter than the period of the corresponding oscillation at the given frequency (T = 1/f) were discarded. (TIF) [file pone.0024893.s001.tif]

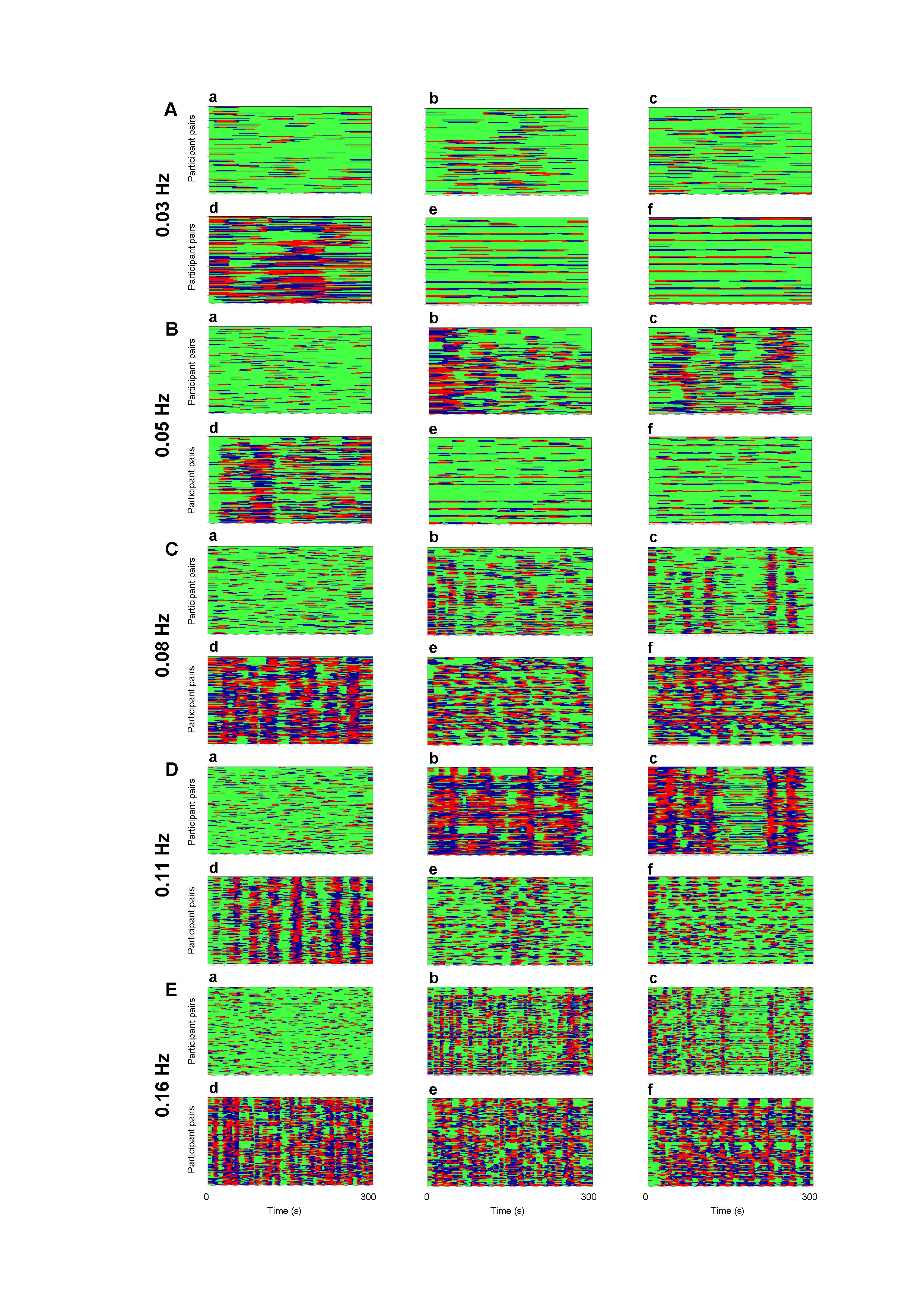

Supplement: Figure S2 — Synchronization patterns of respiration under the different task conditions for the five frequencies of interest. A - E, Frequencies of interest: 0.03, 0.05, 0.08, 0.11, and 0.16 Hz, respectively. a, song singing in unison. b, singing of the song in four parts. c, single canon entry sung in unison. d, canon singing with eyes open. e, canon singing with eyes closed. Each diagram contains 132 lines displaying synchronization pattern of all possible participant pairs in the choir. The phase differences (Δϕ) were color-coded: blue stripes when - π/4 <Δϕ<0; red stripes when 0<Δϕ<+ π/4; and green stripes = non-synchronization when Δϕ< - π/4 or Δϕ> + π/4. (TIF) [file pone.0024893.s002.tif]

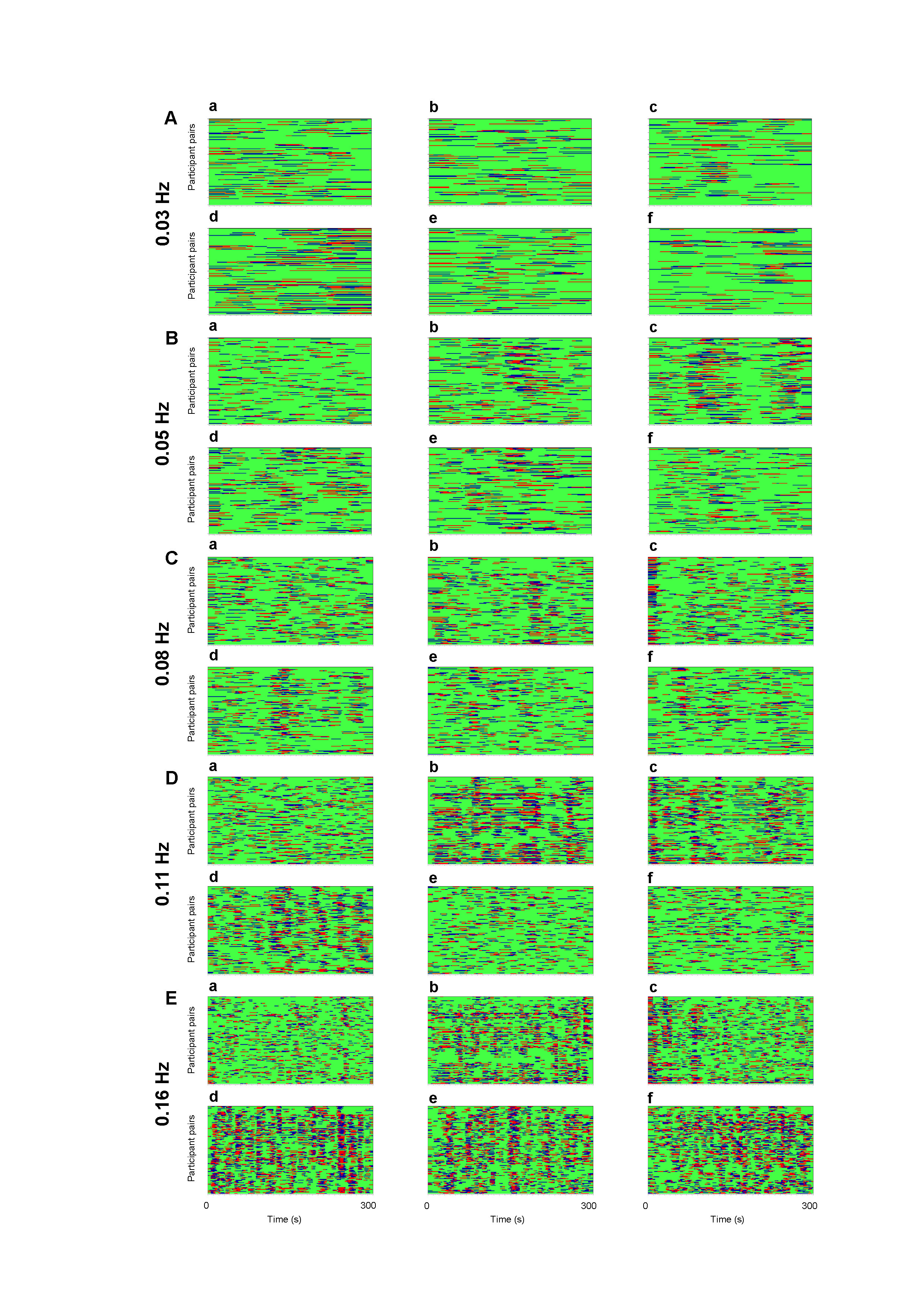

Supplement: Figure S3 — Synchronization patterns of HRV under the different task conditions for the five frequencies of interest. A - E, Frequencies of interest: 0.03, 0.05, 0.08, 0.11, and 0.16 Hz. a, Rest condition; b, Song singing in unison; c, Song with choral singing; d, Canon singing in unison; e, Choral singing of the canon with eyes open; f, Choral singing of the canon with eyes closed. Each diagram contains 132 lines displaying synchronization patterns of all possible participant pairs in the choir. The phase differences (Δϕ) were color-coded: blue stripes when - π/4 <Δϕ<0; red stripes when 0 <Δϕ< + π/4; and green stripes = non-synchronization when Δϕ< - π/4 or Δϕ> + π/4. (TIF) [file pone.0024893.s003.tif]

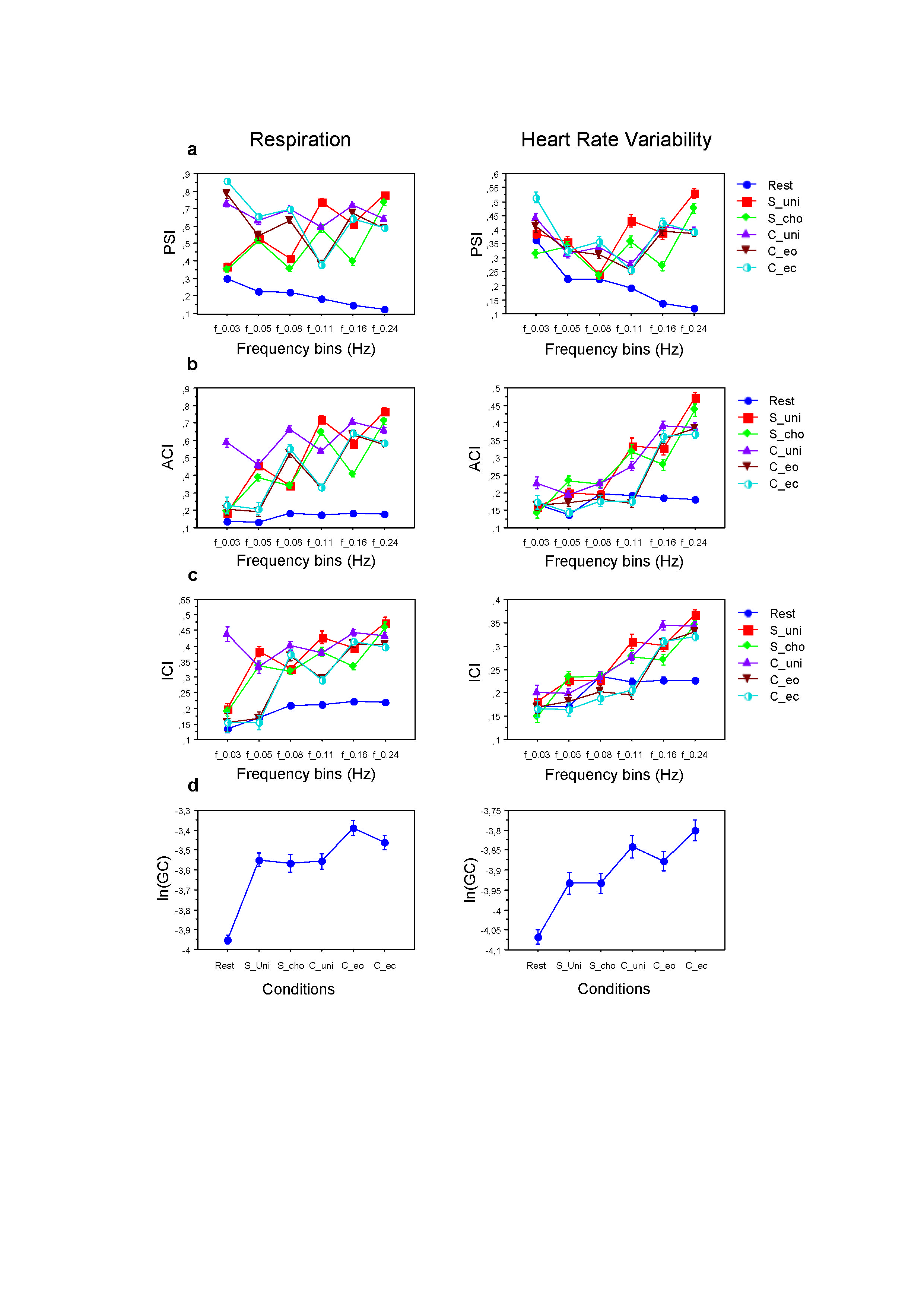

Supplement: Figure S4 — Graphic representation of statistical results. Means and standard error bars of the four synchronization measures (PSI, ACI, ICI, and GC) are displayed for respiration (left column) and HRV (right column) under the six different task conditions (Rest, S_uni, S_cho, C_uni, C_eo, and C_ec) and for the six frequencies of interest (0.03, 0.05, 0.08, 0.11, 0.16, and 0.24 Hz). Note that GC is frequency-independent and was normalized using natural logarithmic transform. (S_uni = singing song in unison; S_cho = singing a four-part song; C_uni = singing canon in unison; C_eo = singing a three-part canon with eyes open; C_ec = singing a three-part canon with eyes closed) (TIF) [file pone.0024893.s004.tif]

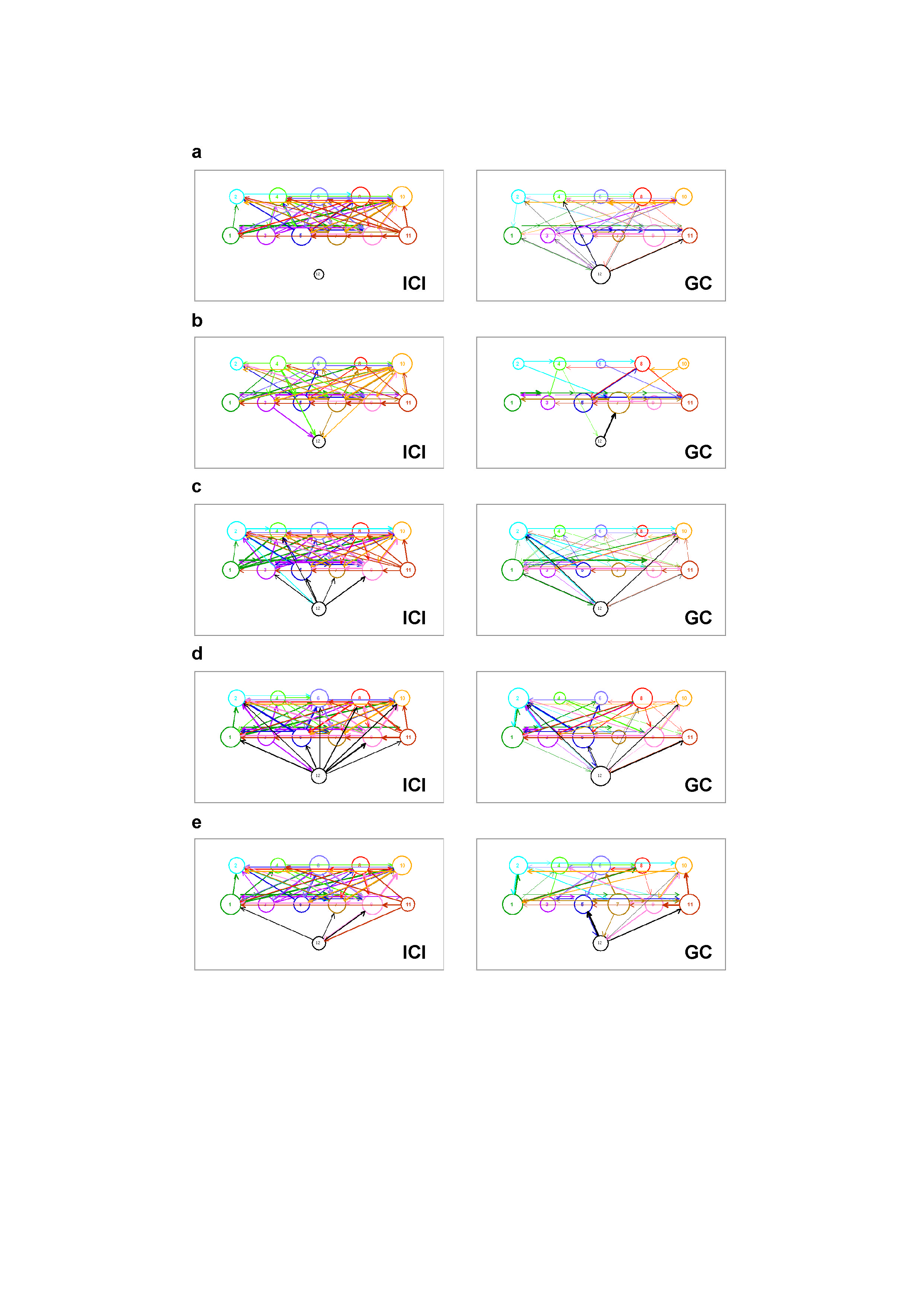

Supplement: Figure S5 — Directed connectivity networks for HRV ICI and GC measures during the different singing conditions. a, singing song in unison. b, singing a four-part song. c, singing canon in unison. d, singing a three-part canon with eyes open. e, singing a three-part canon with eyes closed. ICI-based networks are displayed at the left, and the GC-based networks are displayed at the right. The size of the circle representing choir participants depends on the number of all incoming and outgoing connections. The thickness of the links corresponds to the connection strength, and the arrow displays the direction of the causal influence. Note that the frequency of interest in the case of ICI corresponds to 0.24 Hz and GC is frequency-independent. (TIF) [file pone.0024893.s005.tif]

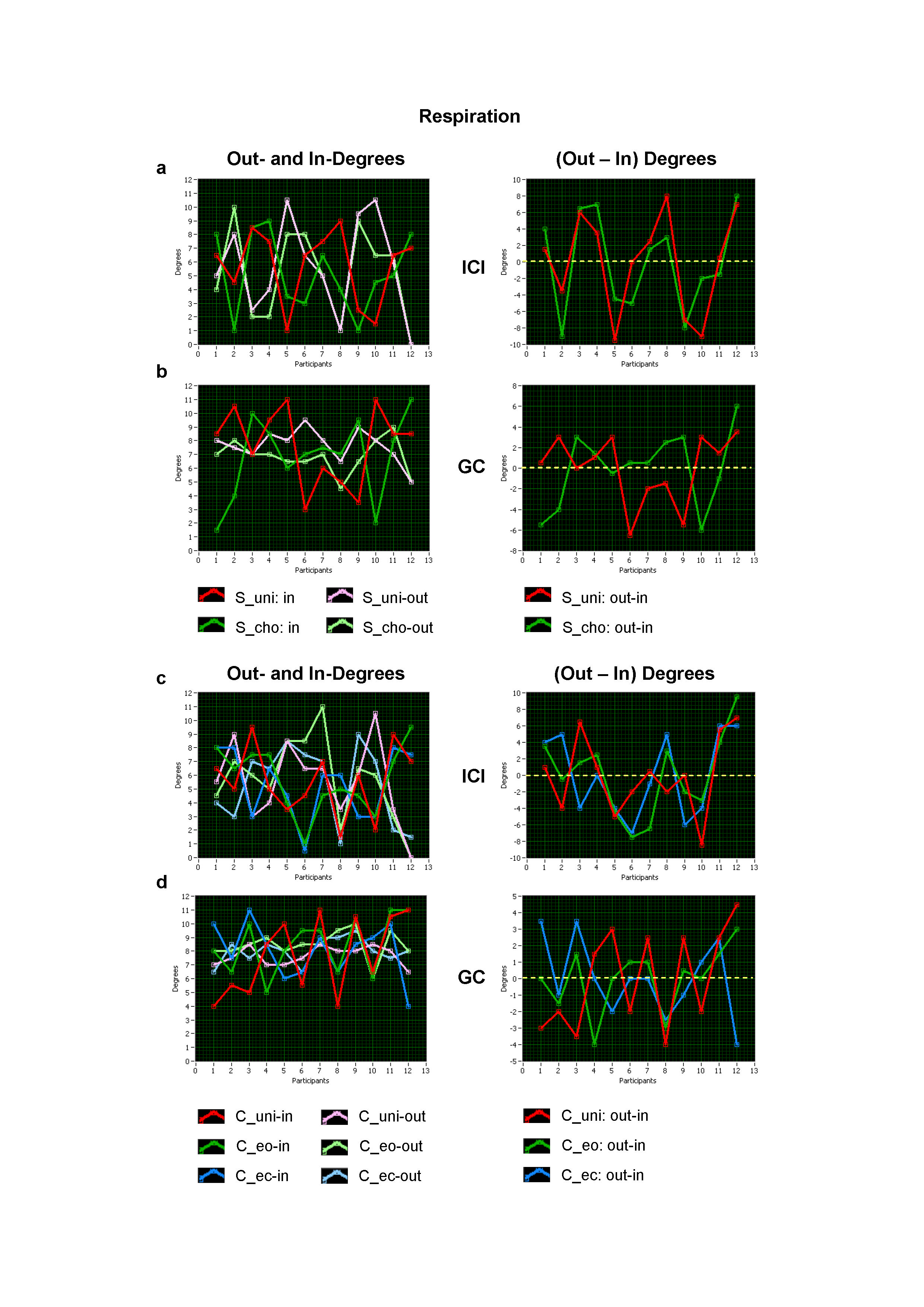

Supplement: Figure S6 — Out- and In-degrees as well as the difference between them for respiration ICI and GC measures during the different singing conditions. a, degrees for ICI measure during song singing. b, degrees for GC measure during song singing. c, degrees for ICI measure during canon singing. d, degrees for GC measure during canon singing. (S_uni = singing song in unison; S_cho = singing a four-part song; C_uni = singing canon in unison; C_eo = singing a three-part canon with eyes open; C_ec = singing a three-part canon with eyes closed) (TIF) [file pone.0024893.s006.tif]

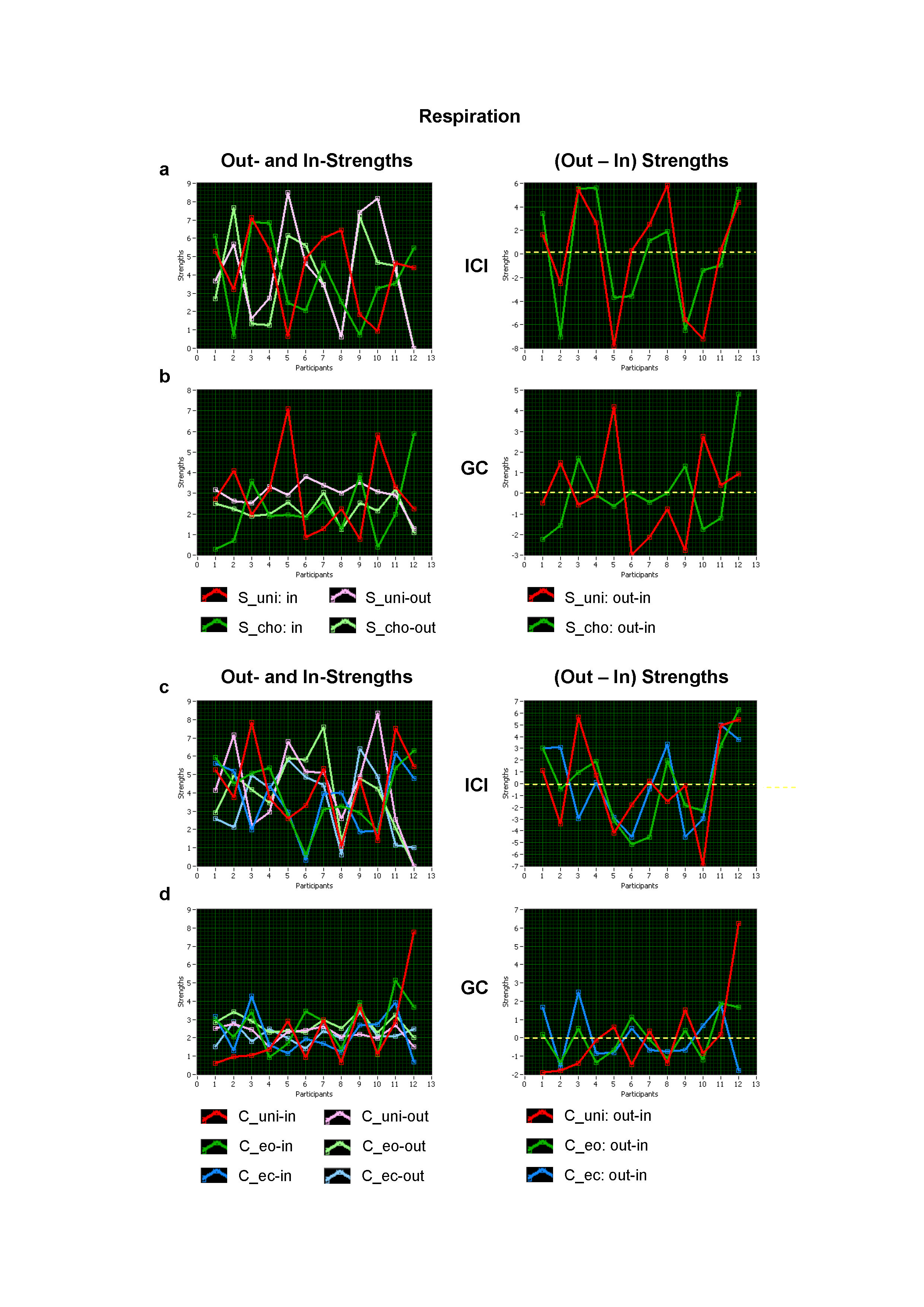

Supplement: Figure S7 — Out- and In-strengths as well as the difference between them for respiration ICI and GC measures during the different singing conditions. a, strengths for ICI measure during song singing. b, strengths for GC measure during song singing. c, strengths for ICI measure during canon singing. d, strengths for GC measure during canon singing. (S_uni = singing song in unison; S_cho = singing a four-part song; C_uni = singing canon in unison; C_eo = singing a three-part canon with eyes open; C_ec = singing a three-part canon with eyes closed) (TIF) [file pone.0024893.s007.tif]

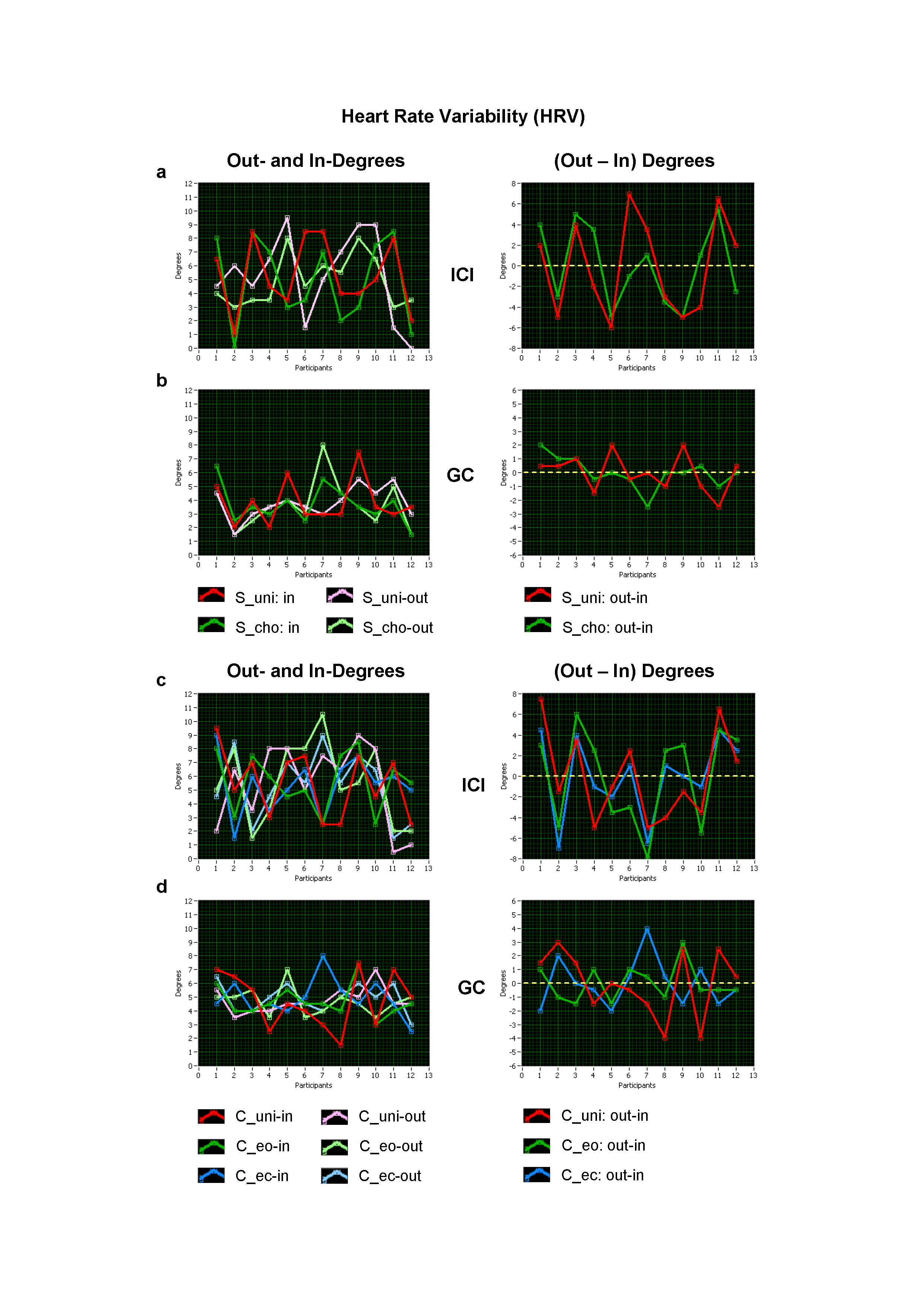

Supplement: Figure S8 — Out- and in-degrees as well as the difference between them for HRV ICI and GC measures during the different singing conditions. a, degrees for ICI measure during song singing. b, degrees for GC measure during song singing. c, degrees for ICI measure during canon singing. d, degrees for GC measure during canon singing. (S_uni = singing song in unison; S_cho = singing a four-part song; C_uni = singing canon in unison; C_eo = singing a three-part canon with eyes open; C_ec = singing a three-part canon with eyes closed) (TIF) [file pone.0024893.s008.tif]

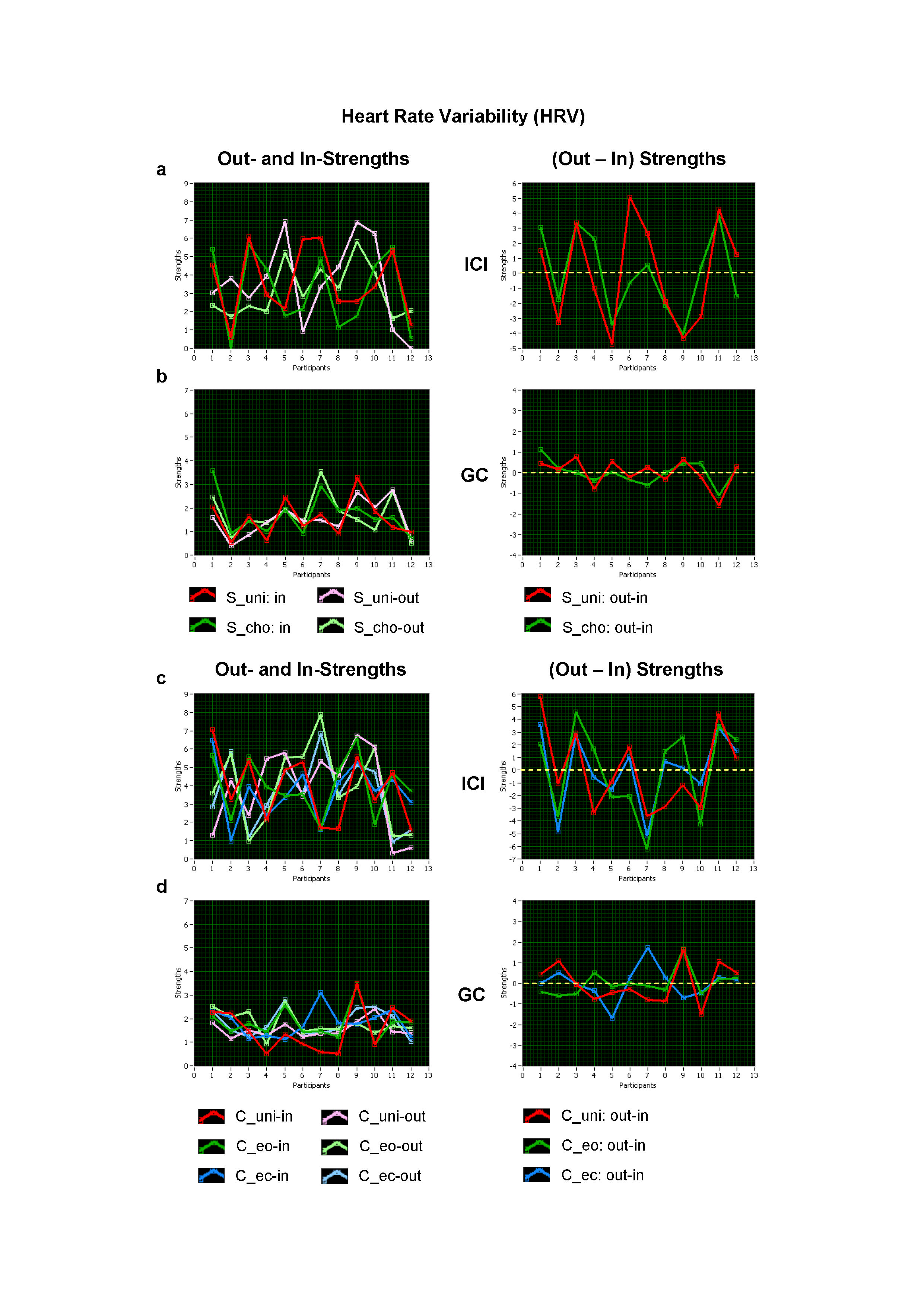

Supplement: Figure S9 — Out- and in-strengths as well as the difference between them for HRV ICI and GC measures during the different singing conditions. a, strengths for ICI measure during song singing. b, strengths for GC measure during song singing. c, strengths for ICI measure during canon singing. d, strengths for GC measure during canon singing. (S_uni = singing song in unison; S_cho = singing a four part song; C_uni = singing canon in unison; C_eo = singing a three-part canon with eyes open; C_ec = singing a three-part canon with eyes closed) (TIF) [file pone.0024893.s009.tif]

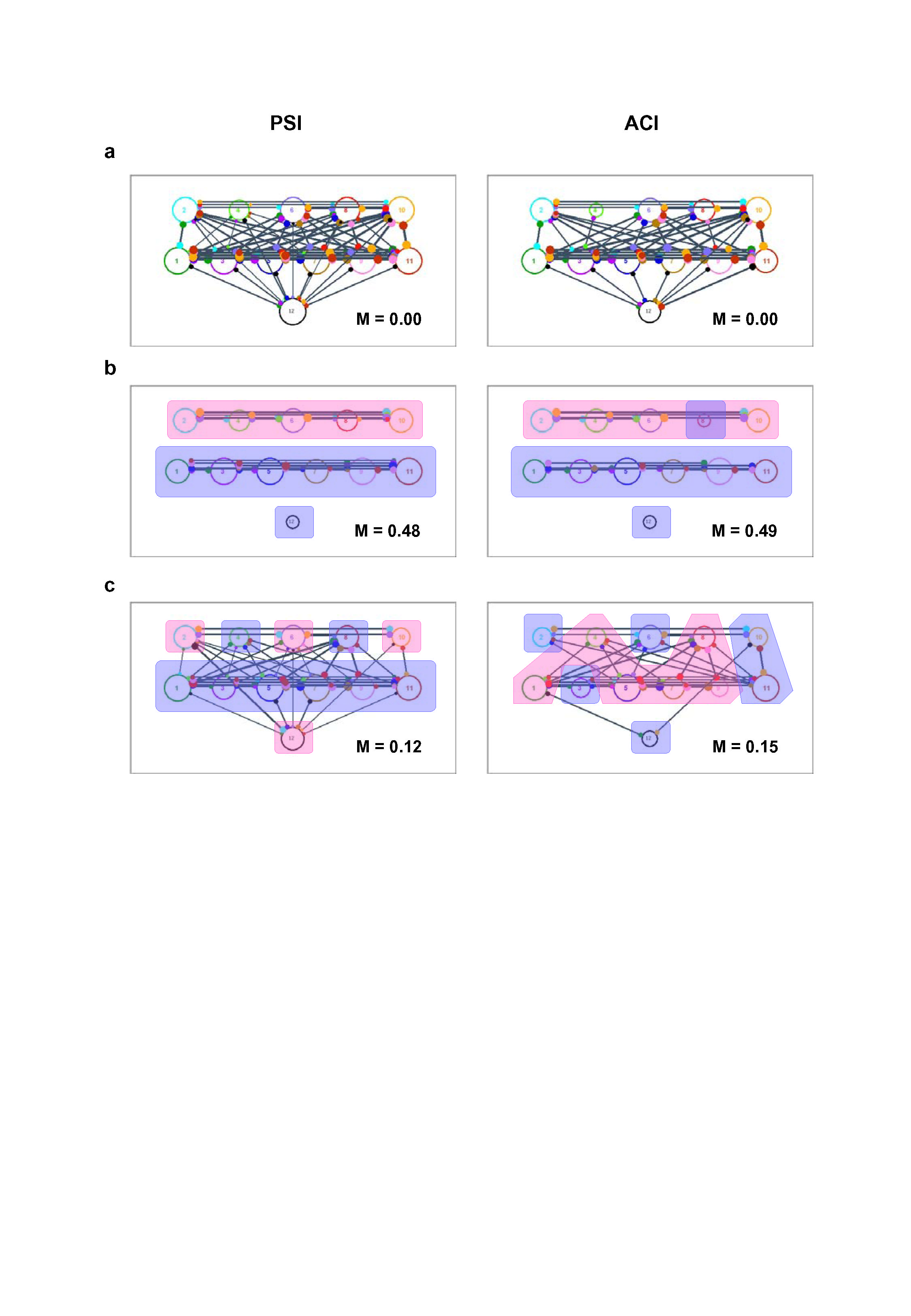

Supplement: Figure S10 — Connectivity networks and modularity effects during singing of the song in the three 100-s consecutive time intervals. PSI and ACI were determined at the moderate frequency of 0.11 Hz. The colored areas display the partition of the networks into modules. a, the first 100-s time interval. The modularity M is zero, and the choir could not be divided in any modules. b, the second 100-s time interval. The choir is strongly divided into two modules separating female and male voices. c, the third 100-s time interval. The modularity M has moderate values, and the female and male voices are mixed in the two modules. (TIF) [file pone.0024893.s010.tif]
