# Supplementary material for: Cardiac and Respiratory Patterns Synchronize between Persons during Choir Singing
Source: PLoS One. 2011 Sep 21;6(9):e24893. doi: 10.1371/journal.pone.0024893 (PMC3177845; doi:10.1371/journal.pone.0024893)
Supplement: Methods S1 — Experimental design and procedure with position of the choir participants and the conductor in the choir during singing as well as the order of test conditions. (DOC) [file pone.0024893.s011.doc]

Supplementary Methods

**Experimental design and procedure**

The institute choir (Max Planck Institute for Human Development, Berlin, Germany) consisting of a conductor and eleven singers (six women and five men) participated in the study. The participants were aligned in a specific position with the singers facing the conductor and standing in two rows.


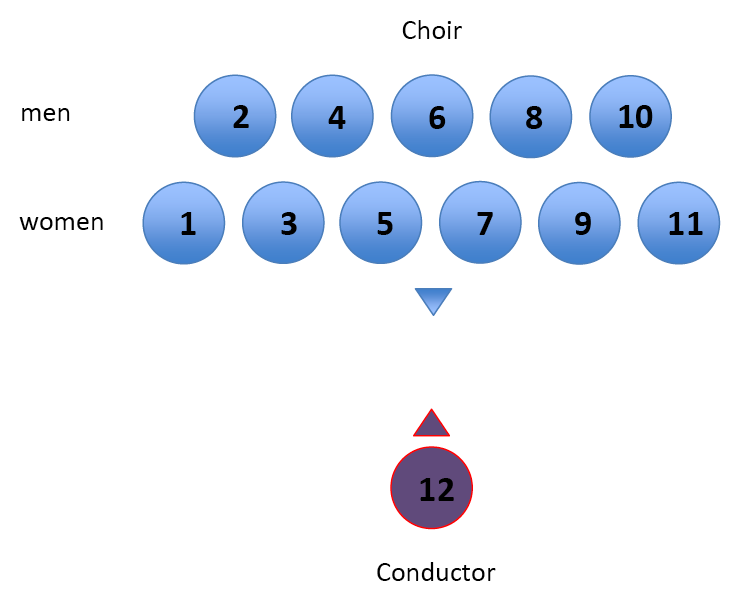


**Position of the choir participants and the conductor in the choir during singing.**

The choir participated in 12 conditions each of which lasted five minutes. The song “Sally Gardens” in D major (Irish Folksong) and the canon “Signor Abbate” in B major (by Ludwig van Beethoven) were performed in different experimental variations. All conditions were carried out in a standing position and lasted about 5 minutes. The order of test conditions was the following:

1. Rest

2. Song singing in unison (all participants sang the soprano part): S_uni

3. Song singing in parts (soprano: participants 1, 3 and 5; tenor: participants 2, 4 and 6; alt: participants 7, 9 and 11; bass: participants 8 and 10): S_cho

4. Song singing in parts (the same voices as in 3): S_cho

5. Song singing in unison (all participants sang the soprano part): S_uni

6. Canon singing in unison (all participants sang the same part): C_uni

7. Canon singing with three individual parts at regular intervals with eyes open (1^st^ part: participants 1, 2, 3 and 4; 2^nd^ part: participants 5, 6, 7 and 8; 3^rd^ part: part: participants 9, 10 and 11): C_eo

8. Canon singing with three individual parts at regular intervals with eyes closed (the conductor sang along a part with the participants 9, 10, and 11): C_ec

9. Canon singing with three individual parts at regular intervals with eyes closed (the conductor sang along a part with the participants 9, 10, and 11): C_ec

10. Canon singing with three individual parts at regular intervals with eyes open (1^st^ part: participants 1, 2, 3 and 4; 2^nd^ part: participants 5, 6, 7 and 8; 3^rd^ part: part: participants 9, 10 and 11): C_eo

11. Canon singing in unison (all participants sang the same part): C_uni

12. Rest
